# Nurse-led fall prevention programs in acute care settings: An integrative review

**DOI:** 10.1016/j.ijnsa.2025.100440

**Published:** 2025-10-25

**Authors:** Sahar Abdulkarim AlGhareeb, Nora Ghalib AlOtaibi, Lujain Adel Sallam, Adnan Innab

**Affiliations:** a Fundamentals of Nursing Department, Imam Abdulrahman Bin Faisal University, College of Nursing, Dammam, Saudi Arabia; b Psychiatric & Mental Health Nursing, Department of Community Nursing, College of Nursing, Imam Abdulrahman Bin Faisal University, Dammam, Saudi Arabia; c king AbdulaAziz University, Medical Surgical college of nursing, Saudi Arabia; d Associate Professor, Nursing Administration and Education Department College of Nursing, King Saud University, Riyadh 12372, Saudi Arabia

**Keywords:** Patient fall, Acute care setting, Fall prevention intervention, Nurse-led, Integrative review

## Abstract

**Background:**

Falls in acute care settings are associated with negative consequences to patients and the healthcare system. Despite growing awareness of the importance of fall prevention in healthcare, there remains a notable lack of comprehensive reviews specifically evaluating nurse-led fall prevention programs in acute care settings.

**Objectives:**

This integrative review aimed to synthesize the current evidence on the nurse-led programs designed to prevent falls among adult inpatients in acute care settings.

**Methods:**

This integrative review has been registered on the International Prospective Register of Systematic Reviews (PROSPERO). The review was guided by Whittemore and Knafl's five-stage integrative review framework. A systematic literature search was conducted across the CINAHL, Scopus, Medline, Web of Science, and ProQuest databases. Only studies published in English between 2016 and 2024, involving adult populations in acute care settings, were included, regardless of geographic location. Three reviewers independently reviewed and assessed the data extraction and methodological quality of each study using the Mixed Methods Appraisal Tool. The results were then analyzed and synthesized through narrative synthesis.

**Results:**

Of 873 articles screened, 23 were included in the review. Four primary themes related to fall prevention strategies have been identified: the fall prevention strategies, nursing training and education, fall rate outcomes, and organizational and environmental factors.

**Conclusion:**

This integrative review highlights the critical role of nurse-led interventions in reducing inpatient falls within acute care settings. Analyzing the key features of these prevention strategies may enable future researchers to enhance and recommend employing multiple intervention strategies for more effective methods for minimizing fall incidents in such environments. Using a single fall prevention strategy demonstrated lower effectiveness than the multiple strategies.

**What is already known**

• Nurse-led prevention programs in acute settings are conducted in several approaches.
• These programs underscore the critical role of nurses in implementing strategies and educating patients to improve patient safety.
• Nurse-led fall prevention programs have not been thoroughly examined within the context of acute care settings.

Alt-text: Unlabelled box

**What this paper adds**

• Nurse-led interventions reduce inpatient falls, though nurses face implementation challenges.
• This review highlighted that studies involved trained nurses in acute care settings who implemented evidence-based strategies, and individualized fall prevention strategies were effective in reducing falls.

Alt-text: Unlabelled box

Falls in hospitals pose a significant patient safety concern and represent a global health challenge (Li & Surineni, 2025). Annually, approximately 1 million patients experience falls during their hospital stay (Li & Surineni, 2025). A fall is an unintentional event where an individual ends up on the ground or against a lower surface (Montero-Odasso et al., 2022). In acute care settings, falls range from one to nine per 1000 patient days (PDs), where 0.4–2.0/1000 PDs of patients experience fall injuries, which indicates one fall every 500-2500 patient days (García-Hedrera et al., 2021). Acute care refers to a setting where individuals receive medical treatment for short but critical episodes of illness. It encompasses various hospital departments, including emergency rooms, trauma care, pre-hospital emergency services, critical care, and other areas requiring urgent attention, such as medical and surgical wards and units (Hirshon et al., 2013).

Falls among inpatients often lead to negative consequences, such as severe injuries and fractures, potentially extending hospital stays and lowering the likelihood of timely discharge (Hill et al., 2019; Ojo & Thiamwong, 2022). A cost analysis conducted over 13 months in two U.S. healthcare systems involving 10,176 patients who experienced falls revealed an average cost of $62,521 per incident, underscoring the substantial financial burden of inpatient falls

(Dykes et al., 2023). The World Health Organization (WHO) recognizes falls as the second leading cause of accidental or unintentional injury deaths worldwide, accounting for approximately 646,000 deaths annually (WHO, 2023).

Numerous researchers reported that falls are considered preventable adverse events (Dykes et al., 2023, 2024; Ojo & Thiamwong, 2022) . To mitigate its risk, it is essential to implement effective fall-prevention measures and strategies. Multidisciplinary collaboration is particularly beneficial in creating a safe, fall-free environment for patients during their hospital stay (Turner et al., 2022). Nurses, as frontline healthcare providers, play a vital role in this initiative by leading educational and preventive programs aimed at reducing the incidence of patient falls (Ojo & Thiamwong, 2022). A meta-analysis of 31 reviewed studies indicated that fall-prevention programs led by, or involving, nursing professionals significantly reduced the incidence of falls by 13% (Orts-Cortés et al., 2024). This is evident through the proactive involvement of nurses in overseeing patient care, offering guidance, and communicating effectively during assessments and while responding to patients’ needs (Ojo & Thiamwong, 2022).

Determining effective strategies to prevent patient falls in hospitals is a critical priority. The world guidelines for fall prevention have recommended implementing different fall prevention strategies, which advocate multifactorial falls risk assessment and personalized interventions to modify falls risks (Montero-Odasso et al., 2022). Several reviews focused on fall prevention across various settings, populations, and healthcare providers (Cameron et al., 2018; Hicks, 2015; Meulenbroeks et al., 2024; Morris et al., 2022; Vlaeyen et al., 2015). For example, a systematic review and meta-analysis examined the role of multi-approach interventions in preventing falls in hospital settings by involving hospital staff (Morris et al., 2022). Other researchers included studies that focused solely on elderly patients to address fall prevention interventions conducted by hospital staff (Cameron et al., 2018). An umbrella review study explored the effectiveness of fall prevention interventions in community settings and residential care groups (Meulenbroeks et al., 2024). Moreover, a systematic review and meta-analysis enrolled thirteen randomized controlled trials that explored the fall prevention programs at nursing homes (Vlaeyen et al., 2015). An integrative review was conducted to examine the effectiveness of rounding as a strategy for reducing falls in acute care settings led by nursing staff. The review included fourteen studies; however, it focused solely on one specific intervention and was conducted a decade ago (Hicks, 2015). Despite the growing awareness of the importance of fall prevention in healthcare, there is a notable lack of comprehensive integrative review studies that specifically evaluate fall prevention programs implemented by nurses in acute care settings. By focusing on the strategies and outcomes associated with these programs, this review highlights the characteristics of the strategy and their effectiveness in improving patient safety and in acute care settings. Specifically, this integrative review aims to synthesize the current evidence on the nurse-led programs designed to prevent falls among adult inpatients in acute care settings.

The following research questions guided the review process:

1- What are the characteristics of nurse-led programs designed to prevent falls among adult inpatients in acute care settings?
2- What is the effectiveness of nurse-led fall prevention programs in reducing falls among adult inpatients in acute care settings?

## 1 Methods

This study was conducted as an integrative review and has been registered on the International Prospective Register of Systematic Reviews (PROSPERO) under the identification number (CRD420251015159). The methodology employed was based on the approach outlined by Whittemore and Knafl (2005), which is well-suited for integrative reviews that incorporate various methodologies, including quantitative, qualitative, and mixed methods studies. This method follows a comprehensive five-stage process to thoroughly address a given issue.

The first stage involves clearly identifying and defining the problem. In the second stage, a literature search is conducted to gather relevant sources and research for foundational understanding. The third stage focuses on evaluating the collected data for validity, reliability, and relevance. In the fourth stage, data analysis uncovers patterns and insights to draw conclusions. Finally, the fifth stage presents the findings, effectively communicating key conclusions and recommendations for future research (Whittemore & Knafl, 2005).

### 1.1 Search strategy

An extensive search was performed using Google Scholar and the SUMMON search engine to reach various databases to find studies, including CINAHL, Scopus, Medline, Web of Science, and ProQuest. Only articles published in English from 2016 through March 2024 were considered. To have rigors and relevant studies, Boolean operators were used, with the search terms that have been adapted for each database: ((TS=(Patient* OR inpatient*) AND TS=("Acute Care" OR "Emergency care" OR "Critical care" OR "Intensive care" OR "Rapid response care") AND TS=("Fall Prevention" OR "Inpatient fall risk management" OR "Hospital fall safety program" OR "Fall risk reduction" OR "Fall safety management" OR "Fall protection" OR "Fall mitigation" OR "Slip, trip, and fall prevention")) AND TS=(nurs*)).

Further searches were carried out on Google Scholar, and all retrieved studies were gathered and organized with Zotero, a reference manager. Limit the search to the English language, open-access articles were selected, while closed-access articles were obtained through affiliated databases.

### 1.2 Eligibility criteria

All included studies must meet the following criteria: (a) qualitative, quantitative, or mixed-method designs; (b) published in full text to ensure transparency, replicability, and fesibility and in English, as translation resources are limited and most relevant literature is published in English; (c) published between 2016 and 2024 as last integrative revirew was conducted in 2015 (Hicks, 2015); (d) specifically address fall prevention strategies led by nurses and their effectiveness and impact on fall rate; (e) explore the organizational and environmental factors regrading nurse-led fall prevention strategies from nurses’ lens; (f) involve adult inpatients in acute care settings (e.g., areas at acute care hospitals, surgical departments, emergency department, intensive care units).

This integrative review excludes the following: (a) non-research articles or tool validation studies; (b) studies with samples consisting of psychiatric or mentally ill patients as their mental status preventing them from comprehending to the instruction; (c) studies addressing fall prevention led by other health care professionals rather than nurses; (d) books, theses, dissertations, conference papers, and performance or quality improvement projects. To ensure consistent methodological rigor and that resources are peer-reviewed and of high quality.

### 1.3 Study selection

All relevant studies were imported to Zotero, a reference manager, and exported to Microsoft Excel, which was utilized to remove duplicates and conduct study screening. The studies were initially screened for title, abstract, and full texts by two independent experts involved in this study, who used separate spreadsheets. Subsequently, both experts independently analyzed the full texts of the studies based on the eligibility criteria. In case of disagreements, a third author stepped in to mediate until a consensus was achieved.

### 1.4 Quality assessment

Two independent reviewers critically appraised the studies using the Mixed Methods Appraisal Tool (MMAT) Version 2018 (Hong et al., 2018). MMAT consists of five appraisal tables depending on the study design. Each appraisal table consists of seven criteria, to which reviewers must respond with ‘yes,’ ‘no,’ or ‘cannot determine,’ and also includes a section for supplementary comments. Of these criteria, only five contribute to the overall score; the initial two criteria, which are standardized across all five appraisal tables, are excluded from the scoring process. These first two criteria evaluate whether the study addresses a salient research question and whether the data collected are sufficient to address that question. If either of these initial criteria receives a ‘no’ or ‘cannot determine’ response, the study is disqualified from further appraisal. The appraisal criteria are provided in Supplementary one following the MMAT scoring criteria (Hong et al., 2018).

The assessment was based on the quantity of “yes” responses. A typical method involves defining quality as a percentage of criteria satisfied, which promotes both appropriateness and transparency. Each “yes” response received one point, and the percentage was calculated; the total score is out of five. The ratings are as follows: 1/5 (20%), 2/5 (40%), 3/5 (60%), 4/5 (80%), and 5/5 (100%). To determine the quality of the studies, high-quality studies meet 80% or more of the criteria, medium-quality studies meet 60%, and low-quality studies meet 40% or less (Supplementary 1).

### 1.5 Data extraction

Data extraction was performed systematically by a single reviewer and independently verified by two additional reviewers to ensure accuracy and transparency. A standardized data extraction form was developed for this purpose. Key information from each study included the authors and publication year, the study's title, design, sample size and characteristics, settings, intervention or program description, and the key findings. Moreover, key findings and the level of evidence were reported.

### 1.6 Data analysis

Three independent reviewers who were involved in the study employed a narrative synthesis in the present study. This method of narrative synthesis is employed to depict similarities and differences across qualitative, quantitative, and mixed-methods findings, utilizing text organized into themes and subthemes, as well as visual aids, to capture patterns, connections, and gaps in the existing literature (Evans, 2003). The effectiveness of the quantitative studies was reported according to the reported results of each study (rate of falls per 1000 patient days /p-values). Additionally, the analysis was cross-validated by the fourth reviewer, who is an expert in the field and involved in the study.

A visual illustration of the primary themes that emerged from this integrative review is presented in Fig. 2. The coding tree was created by the second author using Microsoft Word, based on the synthesis of extracted data, to offer a clearer view of the thematic structure. It was then further refined through discussions among all authors, who provided comments and feedback until the final version was agreed upon.

## 2 Results

### 2.1 Search results

**Fig. 1:**
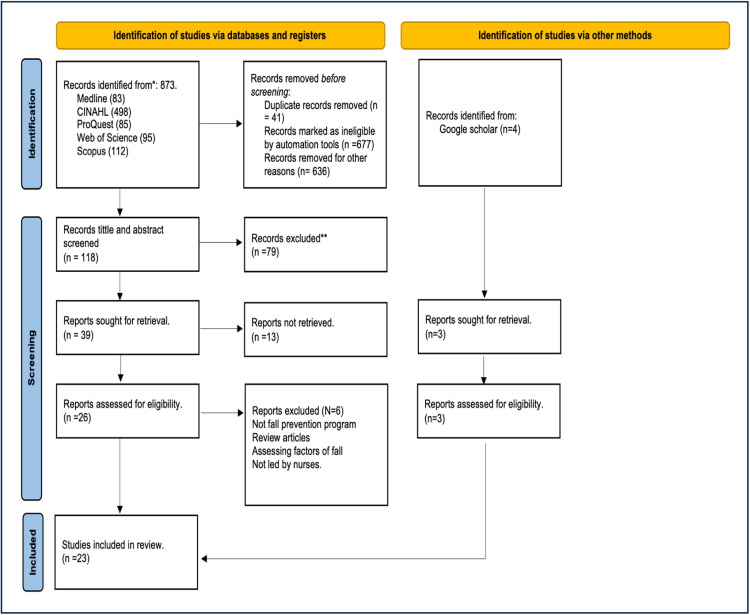
PRISMA 2020 flowchart of the study selection process.

The initial search yielded (n= 873) studies; studies were collected after applying filters and inclusion criteria. duplications (n= 41) were found in CINAHL (n=3), ProQuest(n=10), Medline(n=2), Scopus (n=29), and Web of Science (n=29). After eliminating duplicates, (n= 118) studies were screened based on title and abstract by two independent reviewers who reached a consensus during a meeting with a third reviewer in case of disagreement, (n= 39) were retrieved. Initially, (n=26) studies were included for full-text screening, and (n=20) studies met the inclusion criteria. A complementary search was conducted from Google Scholar, which identified (n= 4) studies, and only (n= 3) studies met the inclusion criteria. Ultimately, (n=23) studies were included for this review. The screening process of the eligibility criteria using the PRISMA 2020 flowchart (Page et al., 2021) is shown in Fig. 1.

### 2.2 Study characteristics

A literature matrix was used to synthesize key data from the included studies. The review encompasses 23 studies (Table 1) published between 2016 and 2024, and involving a total of 728 nurses and 100,354 patients from 92 hospitals worldwide. The studies were conducted across various countries: Australia (n=1), Canada (n=1), China (n=1), Israel (n=2), Saudi Arabia (n=1), Singapore (n=1), Switzerland (n=2), Turkey (n=2), the United Kingdom (n=1), and the USA (n=11). Research designs varied between qualitative (n=7), randomized control trials (n=3), non-randomized control trials (n=4), quantitative descriptive (n=5), and mixed-methods (n=4). Each of the studies aimed to discuss fall prevention strategies led by nurses.

**Table 1:** Characteristics of the Included Studies in the Review.

Author\s, Year of Publication, Country, and Title	Purpose	Methodology	Intervention Description	Measures	Key findings	Limitation	Level of evidence
(Ploeg et al., 2018) (Canada) **Title:** A Sustainability Oriented and Mentored Approach to Implementing a Fall Prevention Guideline in Acute Care Over 2 Years	To assess the impact of a mentored falls prevention guideline implementation focused on enhancing sustainability in reducing fall rates and numbers of serious falls and the experience of participating staff in three acute care hospitals.	**Design:** mix method. **Sample:** n=82 health professionals including (n= 32) nurses. **Setting:** Three community hospitals	Mentored guideline implementation intervention process (NHS Sustainability Model): three interactive workshops and two sustainability action cycles conducted by nursing.	Quantitative: number of falls is obtained from the risk management and patient safety departments. Qualitative: focus group interview.	Quantitative: Two sites reported decrease in falls while one site found increase fall rate in the first quartile The fall s were not significantly different between the three study site (F=2.71, P=.10). For the combined three sites, the mean quarterly fall rate per 1,000 patient days was 6.03 at pre-implementation and 4.98 at postimplementation. The study revealed study sustainability from preintervention to post-intervention related to falls. Qualitative: a major theme was experiences of professional and nonprofessional staff and administrators. The intervention needs refinement and modification overtime.	Low reported number of falls across the sites reduced the statistical power of quantitative analysis. Single-region study reducing generalizability. Aggregated data masking site-specific challenges, incomplete documentation of implementation changes to maintain anonymity of the study staffs. Limited NHS Sustainability Model responses and timepoints.	100%
(Guo et al., 2023) (China) **Title:** Effect of a fall prevention strategy for older patients: A quasi-experimental study.	To explore the effect of a fall prevention strategy on older patients based on the Patient Engagement Framework.	**Design:** A longitudinal quasi-experimental quantitative design. **Sample:** 116 patients from intervention and control groups. **Setting:** Teaching general hospital, from four departments.	The intervention led by nurses from different levels. The research team developed and implemented a personalized fall prevention intervention based on Patient Engagement Framework including five parts: informing, participation, empowerment, cooperation and electronic information support.	Number of falls as counted by nurses. Knowledge-Attitude- Practice (KAP) score. Cronbach's alpha = 0.952 Modified Fall Efficacy Scale score. Cronbach's alpha 0.921	The number of was significantly different between intervention (n=0) to control group (n=3) during the study period.	Non-randomized recruitment of study subjects, Single-center design with limited time/sample size (reduced generalizability), Intervention refinement needed for greater scientific rigor, and Limited outcome measures (lacking secondary indicators like economic benefits).	60%
(Seow et al., 2022) (Singapore) **Title:** Effectiveness of an integrated three-mode bed exit alarm system in reducing inpatient falls within an acute care setting.	To examine the effectiveness of an integrated three-mode bed exit alarm system in reducing inpatient falls within an acute care hospital setting in Singapore.	**Design:** Retrospective before-and-after. **Sample:** 17398 patients. **Setting:** Acute tertiary teaching hospital. Three acute inpatient wards.	Bed exit alarms: The nurse activate the mode of bed alarm if the patient lying on the bed, the activation depends on the nurses' assessment and judgment.	Hospital's electronic database. Hospital's Risk Management System (RMS). Self-reporting to document details of adverse events including falls.	The use of bed exit alarms are associated with a reduction in falls incidence. Preintervention: the incidence of falls was 0.23% (95% CI 0.10% to 0.51%) Postintervention: 0.11% (95% CI 0.05% to 0.25%)	Single-center design, only male gender (though high-volume, may limit generalizability), Low fall rates leading to limited confounder adjustment, Unmeasured nurse compliance with bed exit alarms (potential underestimation of effectiveness), and Lack of standardized guidelines for alarm mode selection (possible differential effectiveness).	80%
(Dykes et al., 2020) (USA) **Title:** Evaluation of a Patient-Centered Fall-Prevention Tool Kit to Reduce Falls and Injuries A Nonrandomized Controlled Trial.	To assess whether a fall-prevention tool kit that engages patients and families in the fall prevention process throughout hospitalization is associated with reduced falls and injurious falls.	**Design:** nonrandomized controlled trial **Sample:** 37231 patients **Setting:** 14 medical units within 3 academic medical centers in Boston and New York City.	A nurse-led tool kit linking evidence-based preventive interventions to patient-specific fall risk factors and designed to integrate continuous patient and family engagement in the fall-prevention process. The patient is given a tailored toolkit according to nurses’ knowledge following their risk assessment. A poster is designed including patient’s risk of fall information and fall prevention plan, which is hanged at patients’ bedside. The poster undergo frequent review with patients and their family at admission and on each shift.	Hospital’s event reporting system.	The study found a 15% decrease in total falls and a significant 34% decrease in falls that resulted in injuries. Overall adjusted 15% reduction in falls postintervention (2.92 falls) compared with preintervention (2.49 falls) per 1000 patient-days, [95% CI, 2.06-3.00 falls per 1000 patient-days].	Uncontrolled variables: Leadership support, communication, timing, and adherence. Limited randomization due to EHR changes and clinician-selected protocols. Single-unit evaluation at two sites (restricts generalizability). Overlapping 95% CIs in secondary analyses (interpret with caution).	100%
(Klaiber et al., 2018) (USA). **Title:** Impact of preoperative patient education on the prevention of postoperative complications after major visceral surgery: the cluster randomized controlled PEDUCAT trial.	To assess the impact of preoperative patient education on postoperative complications and patient-reported outcomes in patients scheduled for elective complex visceral surgery and **(b)** to evaluate the feasibility of cluster randomization in this setting.	**Design:** cluster randomized **Sample:** 244 patients **Setting:** predefined wards of the Department of General, Visceral and Transplantation Surgery, University of Heidelberg.	The trial intervention was a preoperative patient education seminar given by qualified nursing staff one day before surgery.	Hospital database. Serious adverse events (SAE) were documented on specific forms.	The intervention group had significantly lower incidence of fall than control group (0.0% versus 4.2%) of patients respectively, (p = 0.024).	Risk of performance and detection bias in self-reported outcomes (pain, QoL, anxiety, depression), possible contamination effect due to delayed operations/prolonged hospital stays, and Limited generalizability due to monocentric design.	80%
(Wyss-Hänecke et al., 2023) (Switzerland) **Title:** Implementation fidelity of a multifactorial in-hospital fall prevention program and its association with unit systems factors: a single center, cross-sectional study.	To identify ward-level system factors associated with implementation fidelity to a multifactorial fall prevention program (StuPA) targeting hospitalized adult patients in an acute	**Design:** retrospective cross-sectional. **Sample:** 11,827 patients **Setting:** acute care wards at the University Hospital Basel (USB), Switzerland	University Hospital Basel (USB) multifactorial, interdisciplinary fall prevention program. Based on the the Swiss Patient Safety Foundation’s Fall prevention guide, the program consists of fall risk screening, fall prevention interventions, and evaluation of fall events. Trained nurses provide patients with tailored individualized fall prevention intervention.	A 20 items survey was developed by the research team and the validity was confirmed by an expert. No details were given regarding the validation. The hospital’s database and patients’ medical record.	2.8 % (n=336) patients experienced a minimum of one fall, accounting for a total of 491 falls. The fall rate was 5.1 per 1,000 patient days. The study revealed an association between implementation fidelity and number of falls per 1,000 patient days.	Potential recall/social desirability bias in self-reported fidelity data. Variable fidelity, the wards with higher fall rates reported higher fidelity compared to the lower fall rate wards. Lack of patient-level fidelity assessment (aggregated ward-level data only), Possible sampling bias due to routine data use (10% missingness in one variable), Cross-sectional design prevents causal inferences, Confounders were not considered in the measurement as acknowledged by the author. Fall risk tool sensitivity concerns (high false positives/negatives), Dated data (2019) may not reflect COVID-19 impacts, and Context-specific findings, limiting generalizability.	80%
(Innab, 2022) (Saudi Arabia). **Title:** Nurses' perceptions of fall risk factors and fall prevention strategies in acute care settings in Saudi Arabia.	The study aimed to explore nurses’ perceptions of the factors associated with falls and of fall prevention strategies in acute care settings in Saudi Arabia.	**Design:** Cross-sectional, correlational, descriptive study. **Sample:** 102 nurses. **Setting:** A teaching hospital	No intervention. Nurses were asked about their perceptions of the effective fall prevention strategies according to their experience as frontline to patient care.	A survey of injurious fall risk factors and fall prevention interventions, Cronbach's alpha of 0.9. the tool’s name was not mentioned by the author.	Multidisciplinary fall prevention strategies are effective in reducing the prevalence of falls. Nurses enrolled in fall prevention educational program within one year tend to express more awareness of the effective fall prevention strategies (β = −0.364, t = 2.69, r† = 0.290, R2 = 0.084, p < .01) The nurses perceived 10 effective interventions to prevent fall.	Single-site, cross-sectional design limits generalizability and eliminate causation. Convenience sampling may bias results. Context-specific protocols reduce external validity.	60%
(Dykes et al., 2021) (USA) **Title:** Use of a perceived efficacy tool to evaluate the Fall TIPS program.	To assess nurses' opinions of the efficacy of using the Fall TIPS (Tailoring Interventions for Patient Safety) fall prevention program.	**Design:** Survey research. **Sample:** 298 nurses. **Setting:** Seven adult acute-care hospitals in 2 hospital centers located in Boston and NYC.	Three-step Fall TIPS fall prevention program implemented at least for two years.	13-item Fall Prevention EfficiencyScale (FPES). The tool showed proper reliability alpha = 0.83, validity was confirmed.	Nurses perceived the Fall TIPS fall prevention program to be efficacious, they valued the program. The time spent in completing Fall TIPS was less by 10.1 minutes per patient in comparison to other implemented fall prevention programs.	The majority of the participants working on surgical department (57%) leading to limiting representation and generalizability to non-surgical units. Small sample size (298) in seven hospitals at two centers.	60%
(Balaguera et al., 2017) (USA). **Title:** Using a Medical Intranet of Things System to Prevent Bed Falls in an Acute Care Hospital: A Pilot Study.	To conduct a technology evaluation, including feasibility, usability, and user experience, of a medical sensor-based Intranet of things (IoT) system in facilitating nursing response to bed exits in an acute care hospital.	**Design:** (Mixed method) **Sample:** 91 Patients 25 nurses **Setting:** Massachusetts teaching hospital. medical surgical ward.	Sensible Care System. A sensor pad is placed on patients’ beds to sense their movement and send an alert to nurses through a dashboard and their mobile devices. The room flashlight and a high pitch sound both started with patient movement. Nurses are able to see patients’ position time of the alert and can customize the alert setting. Alerts automatically recorded in the system.	Falls were collected from the hospital’s system. Nurses were interviewed in focus groups to identify their experience.	The avarage of nurses response was 49 seconds. The night shift had significantly more time to response (P=.004). Zero bed falls during the study period. Focus groups revealed that nurses found the system integrated well into the clinical nursing workflow and the alerts were helpful in patient monitoring.	Small sample size and single-center design, Recruitment limited to high fall-risk patients, Potential underestimation of effect due to concurrent use of bed alarms, and Possible Hawthorne effect (increased nurse vigilance).	60%
(Weber et al., 2024) (Switzerland) **Title:** Effect of structured nurse-patient conversation on preventing falls among patients in an acute care hospital: A mixed method study	The aim of this study was first to describe the development and use of a patient falls prevention leaflet to be administered by nurses using a structured communication aid, then to analyze both patients’ and nurses’ experiences and opinions regarding its use in clinical practice.	**Design:** mixed method **Sample:** 56 patients 23 nurses. **Setting:** University hospital. Neurological ward.	The hospital follow person-cantered practice framework. The multifactorial fall prevention program guide trained nurses to assess patients risk of fall through completing a checklist. The nurse identify individual safety needs to provide a fall prevention plan. The nurse give the patient a leaflet containing falls information before their individualized structured nurse patient conversation. The plan is reviewed after fall and when patient’s condition changed.	A questionnaire developed by the researcher and reviewed by expert panel.	No overall fall reduction. Regarding nurses completed pre and post survey, their experience and perception to the intervention was not significant, however, they showed a slight higher self-confidence after the conversation which was not significantly different(4.11 ±1.36) to (4.73 ± 1.12), *P*= 0.074. Nurses expressed concerns regarding time constraints and questionable feasibility to the clinical practice.	-A small patient sample due to competing trials and restricted eligibility (excluding cognitively impaired patients and those outside the specialized ward), reducing generalizability. Only nine nurses completed pre and post, and low patient participation as they are enrolled in different studies. these factors lead to decrease in the statistical power. One ward was involved, limiting the generalizability.	60%
(Vechter & Drach-Zahavy, 2021) (Central Israel) **Title:** Effect of nurses' resilience on fall prevention in acute-care hospital: A mixed-methods qualitative study.	To understand the distinctive experience and use of strategies of high- and low-resilience nurses aiming to prevent patient falls.	**Design:** A descriptive mixed method. **Sample:** 24 nurses **Setting:** a medium size hospital. internal wards.	The nurses assessed patients risk of fall using Morse Fall Scale and a fall prevention plan according to the score. The primary investigator conducted a semi structured observation to identify different nurses behaviors in assessment. Followed by immediately interviewing the nurses through semi structured interview.	6-item Brief Resilience Scale, (α = 0.72) Morse Fall Scale (MFS). Semi-structured observations. Semi-structured interview.	One major theme, from maintaining routine to taking control over patients' falls. The low resilience nurses do not do beyond assessing patients by MFS, they believe that falls occurs out of their control. The high resilience nurses do not restrict themselves to the given guidelines and they seek to assessing the reliability and validity of the MFS. They showed more responsibility to spend efforts to prevent falls.	The purposive sampling technique which could lead to a selection bias that affect the study results. Small sample size limit the researcher’s understanding of factors impacting nurses’ resilience.	80%
(Fehlberg et al., 2020) (USA) **Title:** Fall Prevention Decision Making of Acute Care Registered Nurses.	Examine acute care registered nurses’ fall prevention decision-making.	**Design:** qualitative. **Sample:** 12 nurses **Setting:** a tertiary referral medical center. 8 Medical-surgical units.	The nurses were interviewed as 4-phase interactive discussion the following day after they cared of a moderate to high fall risk patient.	Semi structured interviews.	Nine themes developed: fall prevention policy compliance, fear, adequate staffing/patient workload, value of bed alarm, trust, Duty to Preserve Independence, risk versus benefit, nurse judgment, fall prevention activities. Fall prevention policies were helpful.	Single hospital which is not following an evidence based fall prevention program. Only type of units that could differ in prevention than other areas. Moreover, the homogeneity of sample limits the generalizability.	100%
(Radwan et al., 2024) (USA). **Title:** Nurses’ Perceptions of Acute Care Unit Design and Fall Risks.	Understand unit design features that may prevent falls and mitigate patient injuries.	**Design:** Qualitative. **Sample:** 31 nurses. **Setting:** three Veterans hospitals. Medical surgical departments.	The research team walked around inpatient departments and questioned the nurses about how do they think of the wards design and structure and what have been done to change factors led to fall.	Semi-structured focus group interviews.	Nurses’ feedback focused in three categories to prevent patients’ falls; patient rooms, patient bathrooms, unit hallways, and nurses’ stations.	The study was limited to three facilities in the area which limits the generalizability to different settings. The purposive sampling technique which could lead to a selection bias that affect the study results.	100%
(Ayhan Oncu & Seren Intepeler, 2022) (Turkey) **Title:** Nurses’ view of implementation evidence-based fall prevention interventions: A qualitative study	Evaluate nurses’ views of implementation evidence-based fall prevention interventions.	**Design:** descriptive qualitative **Sample:** 17 nurses **Setting:** A training & research hospital. 9 acute care areas.	The participants were involved on Evidence-Based Nursing Practices’ and ‘Patient Falls and Prevention Interventions training program, which was repeated three times. The nurses views about the implemented program were collected.	Semi-structured, in-depth, face-to-face interview.	Three themes were extracted: ‘effectiveness of training program’, ‘barriers’ and ‘suggestions’. The hospital’s evidenced based fall prevention interventions training program was useful, easy, and effective. It contributed to fall prevention and brought benefit to patients and their caregivers.	The study design and single setting limit the generalizability. The views of nurses did not participate were not collected, which may reflect different aspects of thinking, leading to decreases the representation of the study sample.	100%
(Cerilo & Siegmund, 2022) (USA). **Title:** Pilot testing of nurse led multimodal intervention for falls prevention.	Examine the effect of a nurse-led multimodal intervention on hospitalized adults’ levels of fall risk awareness, self-efficacy, and engagement in fall prevention.	**Design:** single-group pre and post-test pilot study. **Sample:** 60 patients **Setting:** A tertiary care hospital. Acute care setting, telemetry and medical-surgical units.	A nurse-led multimodal program was conducted. On pretest; participants attended a video session explaining fall prevention strategies for 10 minutes. The video followed by written and verbal nurses reinforcement which contained information given on the video. Teach back method was used before the post-test. Moreover, the nurses were available all the time for patients support.	Fall Risk Awareness Questionnaire, valid and has an excellent reliability (a = 0.95). Falls Efficacy Scale, High internal consistency (a = 0.83) and high internal validity. Patient Activation Measure, valid and reliable Cronbach’s alpha of (a = 0.87).	The program contributed to a significant enhancement in the awareness toward fall risk among the study participants, (*P* =0.001). No significant difference of the level of engagement before and after the program, however the author reported that this result could be because they were already engaged before the intervention. Fall self efficacy did not show a significant increase (P = 0.271)	There are given factors limiting the generalizability of the study findings, including: The sampling technique, one setting, one group who was highly educated (77%). Moreover, the self-report approach.	80%
(Hoke & Zekany, 2020) (USA) **Title:** Two Sides to Every Fall: Patient and Nurse Perspectives.	To describe and categorize patient and nurse perspectives on falls and nurses’ suggestions for preventing falls.	**Design:** qualitative descriptive. **Sample:** 67 patients **Setting:** cardiac care unit.	The nurses received an education about accountability model. They interviewed the fallen patients to identify their responses to the given questions. The nurses sent theses responses through email.	Three open ended questions through nurse-patient interview.	Three main themes developed: activity (41 falls, 61%), coordination (16 falls, 24%), and environment (10 falls, 15%). The nurses suggest the activation of bed/chair alarm to as a primary fall prevention strategy for the unassisted falls.	Limited areas, the patient cardiac condition increase their risk of fall. The findings cannot be generalized to areas not using bed alarm. Nurses response differently to the alarm.	100%
(Staggs et al., 2020) (USA) **Title:** Unit‐level variation in bed alarm use in US hospitals.	To examine the prevalence of and unit‐level variation in alarm use and estimate the amount of variability in alarm use associated with patient‐ versus unit‐level factors.	**Design:** cross-sectional observational **Sample:** 1,489 patients from 59 units in 57 hospitals from the National Database of Nursing Quality Indicators(NDNQI). **Setting:** University of Kansas Medical Center. Adult medical and surgical units.	Trained nurses assess patients for fall risks and fall prevention strategies including the activation of bed alarms. The nurse is required to document their observations.	NDNQI data collection procedure.	36% of patients used the alarm. The use of alarm varied between units due to different fall prevention strategies, the bed capacity, and previous experience of fall. Patients with previous fall has a 47% chance to use alarm compared to 29% chance for patients did not experience fall. Units with smaller bed capacity had alarm overuse.	The study was limited to NDNQI hospitals which is different in resources, policies and incidence of falls than the non NDNQI hospitals. Additionally, only one unit per hospitals was involved and the participants were self-selected. Moreover, the sample size decided according to the feasibility, not to a power analysis.	80%
(Baris & Seren Intepeler, 2019) (Turkey) **Title:** Views of key stakeholders on the causes of patient falls and prevention interventions: A qualitative study using the international classification of functioning, disability and health.	To explore the views and suggestions of healthcare professionals, patients and family members on the causes of inpatient falls and fall‐ prevention practices.	**Design:** descriptive qualitative. **Sample:** nurses 40% (n=16), patients 20% (n=8), physicians, family members. **Setting:** training and research hospital (acute rehabilitation settings).	No intervention. nurses were interviewed to inform about their views about the effective fall prevention strategies related to their experience.	Semi‐structured interviews.	Regarding fall prevention, four main subthemes developed: related to body function and structure, related to activity and participation, related to environmental factors, and related to personal factors. The implementation of fall prevention programs should reduce hospital’s patients falls.	Factors reducing the generalizability and representation: not all health disciplines were included, the sampling technique, and only four areas were included. Limited sample and limited suggestions for fall prevention.	100%
(Kiyoshi-Teo et al., 2019) (USA) Title: Feasibility of Motivational Interviewing to Engage Older Inpatients in Fall Prevention A Pilot Randomized Controlled Trial.	To determine the feasibility of a brief MI intervention with hospitalized Veterans for fall prevention.	**Design:** 2 arm pilot randomized controlled trial. **Sample:** 67 patients age ≥65 **Setting:** three medical-surgical units at a Veterans Affairs hospital.	Brief motivational interviewing (MI) intervention based motivation guide. Open ended questions.	Mon- treal Cognitive Assessment-Basic Modified Falls Behavioral [M-FaB] Scale, Cronbach’s alpha (base- line during hospitalization) was 0.76 and the 29-item M-FaB ranged from 0.70 to 0.74 during 2-day.	The intervention is feasible, affective, and appropriate for the clinical practice. Patients demonstrated fall prevention behavior during hospitalization. The fall prevention behavior was significantly different between groups (M-FaB, *p* = 0.031) The MI intervention did not significantly reduce the actual fall rate during the study. Density of falls during the 3-month follow-up period was 26.1% higher for the intervention arm. The incident density for falls for the con- trol arm was 0.15 (95% confidence interval [CI] [0.057, 0.243]), and 0.18 for the intervention arm (95% CI [0.072, 0.307]). However, this difference was not significant.	Patients’ age and health status limited their capability to participate. Limited generalizability (single center, small sample, only male highly oriented male patients). The high attrition, and the design was unblinded. the control group showed high fall prevention behavior.	60%
(Timmons et al., 2019) (United Kingdom) **Title:** Trialling technologies to reduce hospital in-patient falls: an agential realist analysis.	To examine the fall and its prevention as a situated phenomenon of knowledge that is made and unmade through intra-actions between environment, culture, humans and technologies.	**Design:** qualitative. **Sample:** 20 nurses and Health Care Assistants, and 18 patients. **Setting:** hospital ward in acute hospital.	Pressure sensors system. Structured observations to patients’ location and activity, sensor alarm activation and nurses’ response to the pager pleep and any action was seen.	Semi-structured interviews.	The nurses did not response to the alarm as intended due to technical challenges, shortage of staffs, stressful workload. It is a waste of clinical time. Nurses did not agree about the accuracy of the sensor in addressing patients at risk of fall. Some false alarm incidence. It restricted patients’ movement to avoid false alarm.	Technical challenges. the alarm was not used as intended. The heterogeneity of the nurses level of seniority affected their response to the sensor notifications. The senior nurses showed more level of enthusiasm for the system.	100%
(King et al., 2018) (USA) **Title:** Impact of Fall Prevention on Nurses and Care of Fall Risk Patients.	Explore nurses’ experiences with fall prevention in hospital settings and the impact of those experiences on how nurses provide care to fall risk patients.	**Design:** qualitative. **Sample:** 27 registered nurses and nursing assistants. **Setting:** two teaching hospitals. Adult Medical surgical units.	No intervention. Nurses stated they do not have fall prevention protocol at their units. As a standard routine, to reduce fall, nurses are instructed to identify high risk patients, to applay bed/chair alarm, and to response to the alarm.	In-depth one-to-one interviews. Unstructured open-ended questions.	To prevent fall; nurses fall prevention strategies varied depending on the incidence of fall at their units. Units with high incidence of fall: restrict high risk patient movement. Units with low incidence of fall tend to strengthening the patients and provide safe mobility using assisted people or equipment.	The sampling technique and observation behind interviews may affect the results and limited generalizability to similar settings.	100%
(Barker et al., 2016) (Australia) **Title:** 6-PACK programme to decrease fall injuries in acute hospitals: cluster randomised controlled trial.	To evaluate the effect of the 6-PACK programme on falls and fall injuries in acute wards.	**Design:** Cluster randomized controlled trial. **Sample:** 31411 patients. **Setting:** 24 acute wards from 6 Australian hospitals.	Patients were assigned into usual care group or the 6-PACK programme included a fall risk tool and individualized use of one or more of six interventions: “falls alert” sign, supervision of patients in the bathroom, ensuring patients’ walking aids are within reach, a toileting regimen, use of a low-low bed, and use of a bed/chair alarm.	Patients’ medical records. Verbal reports from the ward nurse unit manager. Monthly audit of hospital incident reports. Administrative databases.	A total of 1831 falls occurred in 12 months. No significant difference between the intervention and control groups in fall rates (incidence rate ratio 1.04, 0.78 to 1.37; P=0.796).	Convenient sampling limits the representation of the study sample. The study was not blinded this could raise the risk of bias, showing larger intervention’s effect.	100%
(Vechter & Drach-Zahavy, 2024) (Israel) Effect of Nurse Proactive Behavior on Patient Education for Fall Prevention in Acute Settings: A Moderated-Mediation Model.	To develop and examine a moderated- mediation model for proactivity in preventing patient falls.	**Design:** cross-sectional observational **Sample:** 101 nurses & 271 patients. **Setting:** 14 acute care adult wards.	Nurses provide education to patients regarding fall, based on Joint Commission International Accreditation and the hospital’s manual.	Brief Resilience Scale (BRS), good reliability (α=0.70) Proactive behaviors were assessed with a three-item scale, good reliability (α=0.70). Social capital, excellent reliability, (α=0.85). Structure interview with patients.	A significant positive correlation was found between nurse proactivity and adherence to procedures (r = 0.41, p < 0.01). No significant correlation between nurses resilience and proactivity with patient education about fall prevention (p > 0.05).	The study design limits the identification of a causation. The used of self reported questionnaire. the observation used could affect the participants’ natural behavior. convenient sampling and one setting limited the generalizability.	80%

### 2.3 Methodological quality

Two reviewers independently critically appraised the included studies as shown in (Table 2). The included study revealed a good level of evidence ranging between 60% and 100%. Studies obtained 100% (n=10), 80% (n=7), and 60% (n=6), where the majority of the studies showed a high quality of evidence (Supplementary 1).

**Table 2:** The Critical Appraisal Tool of the Included Studies.

### 2.4 The study themes

**Fig. 2:**
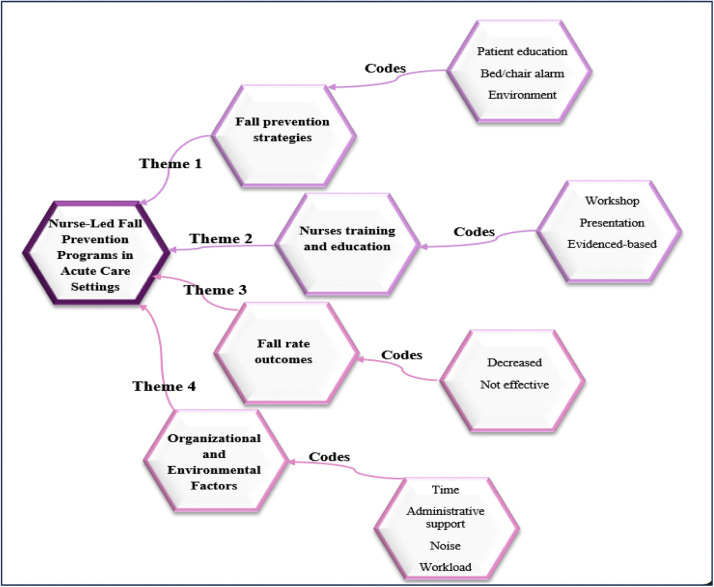
The Coding Tree of the Study Themes.

In this integrative review, four primary themes have been identified. In relation to the first research question, two themes have emerged as fall prevention strategies and nurses' training and education, which addressed the characteristics of the fall prevention program. While fall rates, outcomes, and organizational/environmental factors answered the second research question, which addressed the effectiveness of the nurse-led fall prevention programs in reducing falls (Fig. 2).

#### 2.4.1 Fall prevention strategies

In this integrative review, eleven studies implemented evidence-based strategies. The Swiss Patient Safety Foundation’s Fall Prevention Guide (Wyss-Hänecke et al., 2023), Evidence-based fall prevention intervention(Ayhan Oncu & Seren Intepeler, 2022), Motivational Interview-Based Communication Guide for Fall Prevention (Kiyoshi-Teo et al., 2019), 6-PACK programme (Barker et al., 2016), fall prevention based on Joint Commission International Accreditation, hospital standards(Vechter & Drach-Zahavy, 2024), National Health Service sustainability model (Ploeg et al., 2018), toolkit linking evidence-based preventive interventions (Dykes et al., 2020), person-centered practice framework, and the multifactorial fall prevention program (Weber et al., 2024), Recognition-Primed Decision Model and the Quality Health Outcomes Model (Fehlberg et al., 2020), nurse-driven accountability model (Hoke & Zekany, 2020), and the Patient Engagement Framework(Guo et al., 2023). The characteristics of these evidence-based strategies are shown in (Table 3.)

**Table 3:** Studies implemented fall prevention strategies based on evidence-based Strategies.

Author & study Title	The Evidenced based strategies	Characteristics of the evidenced based Strategies
(Wyss-Hänecke et al., 2023)	The Swiss Patient Safety Foundation’s Fall Prevention Guide	It is four steps multifactorial interventions that is applied through: patient identification, patient risk assessment, intervention implementation, and evaluation of the implemented intervention.
(Dykes et al., 2020)	Toolkit linking evidence-based preventive interventions	The Fall Tailoring Interventions for Patient Safety (TIPS) toolkit, as described in Dykes et al. (2020), is an evidence-based hospital fall prevention program led by nurses. It involves three steps: assessing each patient's fall risk, developing a personalized prevention plan, and applying it with effective communication. The toolkit creates tailored interventions like supervision, environmental changes (bed rails, low beds, non-slip socks), and education, encouraging patient and family participation to improve adherence. Available in paper and electronic formats, it is integrated into clinical workflows, involving the care team, patient, and family, to promote personalized, collaborative care. Fall TIPS has significantly reduced inpatient falls.
(Guo et al., 2023)	Patient Engagement Framework	An evidence-based patient engagement framework for fall prevention is based on a partnership model that initiates with a customized risk assessment to inform patients about their particular risks. This facilitates collaborative goal-setting and shared decision-making, empowering patients and enhancing their self-efficacy. Tailored, multimodal interventions—such as exercise regimens or modifications to the home—are subsequently carried out according to the patient’s degree of engagement. The approach actively incorporates caregivers and family members for support and relies on continuous follow-up to reinforce progress and address any challenges that may arise.
(Ayhan Oncu & Seren Intepeler, 2022)	Evidence-based fall prevention intervention	This evidence-based intervention is based on the Hospital Service Quality Standards, which were determined by the Ministry of Health of Turkey. The nurses are required to recognize risk factors, indicate the use of high fall risk markers, and educate the patient and the caregivers about falls with the patient.
(Kiyoshi-Teo et al., 2019)	Motivational Interview-Based Communication Guide for Fall Prevention	A communication guide based on motivational interviewing (MI) for fall prevention helps individuals recognize and commit to strategies that reduce falls by utilizing Open-ended questions, Affirmations, Reflections, and Summaries (OARS). This approach includes showing empathy, highlighting the gap between current habits and desired outcomes, adapting to resistance, and encouraging self-efficacy to inspire internal motivation for change.
(Barker et al., 2016)	6-PACK programme	The "6-Pack Program" for Fall Prevention, based on evidence and executed by Barker et al. (2016), involved a structured set of six essential interventions for all patients identified as high-risk: a fall risk alert sign on the door, a scheduled two-hour regimen for toileting and rounding, non-slip footwear, functional bed/chair alarms, personalized education for patients and their families, and a customized care plan. This comprehensive strategy simultaneously addressed environmental, physiological, and behavioral risk factors by implementing uniform protocols, enhancing staff vigilance, promoting safe mobility, and actively involving patients and their families in the prevention efforts. As a result, this methodology contributed to a significant and sustained decrease in both the incidence of falls and fall-related injuries.
(Vechter & Drach-Zahavy, 2024)	Fall prevention based on Joint Commission International Accreditation, hospital standards	Based on Joint Commission International (JCI) accreditation standards, hospital leadership is responsible for integrating this data-driven program into the facility's core quality and safety objectives, ensuring that resources and policies are aligned to minimize fall risks and prevent harm. To prevent falls effectively, hospitals must implement a comprehensive strategy that begins with a risk assessment for each patient using a validated tool upon admission and regularly thereafter. For high-risk patients, a tailored prevention plan should include evidence-based measures such as bed alarms, non-slip footwear, scheduled rounding, and environmental modifications. Ongoing staff training, patient and family education, and a non-punitive culture that encourages incident reporting are crucial for improvement. Ultimately, hospital leadership is responsible for integrating this data-driven approach into quality and safety objectives to reduce fall risks and prevent harm.
(Ploeg et al., 2018)	National Health Service sustainability model (NHS)	The NHS model serves as a diagnostic tool designed to evaluate the effectiveness of a fall prevention strategy. (1) Assessment: Healthcare teams utilize the model to review their implementation strategies across various components. (2) Score Calculation: The model generates scores, with initial low scores in specific areas being a common occurrence early in the project. (3) Action Planning: It encourages teams to pinpoint aspects needing improvement and to act promptly to enhance future sustainability. (4) Mainstream Integration: Ultimately, the aim is for the new practice to become ingrained in daily operations, making it standard procedure that can endure over time.
(Weber et al., 2024)	Person-centered practice framework, and the multifactorial fall prevention program	Weber et al. (2024) employed a person-centered practice framework through structured nurse-patient discussions complemented with a multifactorial fall prevention program aimed at reducing falls amongst patients in an acute care hospital. Upon admission, nurses performed individualized risk assessments followed by a patient-initiated discussion regarding the fall risk factors specific to the patient. Education was included in the conversation using a structured leaflet that discussed the following topics: what it means to prevent falls, designing safe activities to prevent falls, and providing the patient with an individualized risk assessment according to their accomplishments in the education session. Educating the patient on important issues surrounding fall risk and promoting shared decision-making demonstrated the patient's contributions to developing a personalized safety plan.
(Fehlberg et al., 2020)	Recognition-Primed Decision Model and the Quality Health Outcomes Model	It is a framework that integrates the Recognition-Primed Decision (RPD) Model with the Quality Health Outcomes Model (QHOM) to enhance fall prevention strategies. The RPD Model highlights how nurses utilize their experience and intuition to identify patient instability and environmental risks, leading to immediate actions such as bedside support or alarms. Meanwhile, the QHOM connects interventions to outcomes by considering patient attributes and system-level strategies, ensuring proactive and adaptable fall prevention measures. This combined approach emphasises the link between clinical judgment and evidence-based thinking, resulting in a significant reduction in falls.
(Hoke & Zekany, 2020)	Nurse-driven accountability model	A nurse-driven accountability model is a framework for professional practice that empowers nurses to take ownership of their decisions and actions to improve patient care outcomes. By decentralizing decision-making and fostering a culture of ownership, the model shifts responsibility from top-down management to the nurses at the point of care. This model is implemented through key priniciples: (1) empowring nurses by giving them the authority to decide, (2) engage nurses in the fall prevention strategies, (3) clear expectations by providing accessable standars and goals for all, (4) shared responsibility, that all health providers are responsible about patient saftey, and (5) contineous learning, it allow nurses to further learn from their mistakes rather than blaming them.

Three of these studies involved patients in developing individualized fall prevention strategies based on their assessment and needs (Dykes et al., 2020; Guo et al., 2023; Wyss-Hänecke et al., 2023). These studies include a randomized control trial, a quasi-experimental design, and a cross-sectional design. Fall prevention programs were delivered to patients through the provision of educational materials (Dykes et al., 2020; Guo et al., 2023; Ploeg et al., 2018; Weber et al., 2024). These materials included a fall prevention guidance manual, leaflets, color cards, and bedside displays. These randomized controlled trials, quasi-experimental design, and a prospective longitudinal study demonstrated that nurses were trained in an evidence-based approach (Dykes et al., 2020; Guo et al., 2023; Ploeg et al., 2018). Finally, only two studies—a cross-sectional observational study and a mixed-method study indicated that enhancing nurses’ resilience and proactive approach can lead to more effective patient education on fall prevention. However, nursing proactivity did not have a significant impact on patient education (p > 0.05) (Vechter & Drach-Zahavy, 2021, 2024).

Furthermore, two additional experimental studies implemented fall prevention strategies through video presentations, followed by reinforcement of the information conveyed (Cerilo & Siegmund, 2022; Guo et al., 2023). Moreover, a nurse-patient structured conversation strategy was used to educate patients about fall prevention. This approach was reported in four studies with different designs: a randomized controlled trial, a mixed-methods study, and a qualitative study (Fehlberg et al., 2020; Klaiber et al., 2018; Vechter & Drach-Zahavy, 2024; Weber et al., 2024).

Other studies implemented multiple fall prevention strategies. Eight of these studies used bed/chair alarms (Balaguera et al., 2017; Barker et al., 2016; Fehlberg et al., 2020; Hoke & Zekany, 2020; King et al., 2018; Seow et al., 2022; Staggs et al., 2020; Timmons et al., 2019). Furthermore, three studies highlighted the importance of adjusting standard environmental strategies, such as ensuring side rails are raised, maintaining floor integrity, keeping hallway clear, providing patients with accessible call bell, considering nurses’ judgment when selecting appropriate fall prevention measures, instructing patients to call for assistance when ambulating, and reducing toilet privacy when necessary (Fehlberg et al., 2020; Guo et al., 2023; Radwan et al., 2024).

#### 2.4.2 Nursing training and education

Staff training played a critical role in seven studies, employing diverse methods to equip nurses for effective implementation of fall prevention programs (Ayhan Oncu & Seren Intepeler, 2022; Dykes et al., 2020; Guo et al., 2023; Hoke & Zekany, 2020; Ploeg et al., 2018; Weber et al., 2024; Wyss-Hänecke et al., 2023). A randomized controlled trial found that nurses participating in a training program focused on customizing the fall prevention toolkit experienced a 15% overall decrease in patient falls (Dykes et al., 2020). Two studies examined the training of nurses to facilitate conversations with patients about fall prevention strategies (Hoke & Zekany, 2020; Weber et al., 2024). Despite methodological differences, a strong consensus emerged across both studies’ findings that fall rates decreased following patient involvement in such conversations. Formal and informal fall prevention training enhanced nurses’ confidence and adherence to fall prevention protocol (Guo et al., 2023; Klaiber et al., 2018; Ploeg et al., 2018; Seow et al., 2022; Staggs et al., 2020; Wyss-Hänecke et al., 2023).

#### 2.4.3 Fall rate outcomes

A total of seven studies indicated that the occurrence of falls was notably decreased through the implementation of different fall prevention methods. These methods encompass bed and chair alarms, programs based on evidence for fall prevention, and individualized interventions based on assessments conducted by nurses (Balaguera et al., 2017; Dykes et al., 2020; Guo et al., 2023; Klaiber et al., 2018; Ploeg et al., 2018; Seow et al., 2022; Wyss-Hänecke et al., 2023). Across three studies, there was a notable reduction in the occurrence of falls, ranging from 15% to more than 35% (Dykes et al., 2020; Guo et al., 2023; Wyss-Hänecke et al., 2023).

However, six additional studies indicated that the fall prevention strategies used were ineffective, even when employing various strategies. These strategies comprised a bed and chair alarm, individualized fall prevention plans, and educational methods for patients (including video, motivational interviewing, and structured conversations between nurses and patients) (Barker et al., 2016; Cerilo & Siegmund, 2022; Kiyoshi-Teo et al., 2019; Ploeg et al., 2018; Staggs et al., 2020; Weber et al., 2024).

#### 2.4.4 Organizational and environmental factors

Thirteen studies discussed nurses’ perceptions regarding nurse-led fall prevention strategies (Ayhan Oncu & Seren Intepeler, 2022; Balaguera et al., 2017; Baris & Seren Intepeler, 2019; Dykes et al., 2021; Fehlberg et al., 2020; Hoke & Zekany, 2020; Innab, 2022; King et al., 2018; Ploeg et al., 2018; Radwan et al., 2024; Timmons et al., 2019; Vechter & Drach-Zahavy, 2021; Weber et al., 2024). Of these five studies, nurses found that using bed and chair alarms was effective for fall prevention (Balaguera et al., 2017; Fehlberg et al., 2020; Hoke & Zekany, 2020; King et al., 2018; Timmons et al., 2019). However, some nurses expressed challenges with this strategy, such as increased workload and technical issues (Balaguera et al., 2017; Fehlberg et al., 2020; King et al., 2018; Timmons et al., 2019). Across five studies, it was found that nurses consider modifying the environment to be an essential strategy for fall prevention. This includes measures such as assigning nurses to patients located nearby with clear hallways and ensuring that call bells are within easy reach (Ayhan Oncu & Seren Intepeler, 2022; Baris & Seren Intepeler, 2019; Fehlberg et al., 2020; Hoke & Zekany, 2020; Radwan et al., 2024).

Furthermore, four studies reported that nurses perceived the effectiveness of fall prevention strategies to be closely associated with their education and training (Ayhan Oncu & Seren Intepeler, 2022; Innab, 2022; Ploeg et al., 2018; Radwan et al., 2024). However, nurses indicated that limited time and busy clinical schedules were common challenges to participating in training and education (Ploeg et al., 2018; Weber et al., 2024).

Various studies have indicated that training nurses to involve patients and family members in fall prevention strategies recognizes their role as key organizational stakeholders in patient safety. Notably, involving families in patient education and prevention strategies makes fall prevention strategies more effective (Ayhan Oncu & Seren Intepeler, 2022; Baris & Seren Intepeler, 2019; Dykes et al., 2021; Ploeg et al., 2018).

Also, nurses involved emphasized the significance of conducting early patient assessments to identify the level of fall risk, to tailor appropriate fall intervention strategies based on each patient's condition (Baris & Seren Intepeler, 2019; Fehlberg et al., 2020; Innab, 2022). In four studies, nurses emphasized the critical role of multidisciplinary collaboration in the implementation of fall prevention strategies (Ayhan Oncu & Seren Intepeler, 2022; Innab, 2022; Ploeg et al., 2018; Radwan et al., 2024). Across five studies, nurses expressed that administrative support plays a crucial role in the success of fall prevention strategies through effective communication, providing adequate resources, and maintaining sufficient nurse staffing (Ayhan Oncu & Seren Intepeler, 2022; Fehlberg et al., 2020; King et al., 2018; Ploeg et al., 2018; Radwan et al., 2024).

## 3 Discussion

This integrative review synthesizes findings on nurse-led fall prevention programs in acute care settings. Four primary themes related to fall prevention strategies have been identified: fall prevention strategies, nursing training and education, fall rate outcomes, and organizational and environmental factors. The review included 23 studies from various countries, highlighting the potential global significance of the findings in addressing patient falls. The quality of evidence ranged from moderate to strong, supporting the validity of the conclusions for nursing research and practice.

The nurse-led fall prevention programs were found to vary in their approaches, including the use of bed and chair alarms, environmental modifications, and various patient education strategies. This review also revealed inconsistent findings across studies regarding the effectiveness of bed and chair alarms in reducing falls. Studies conducted by Balaguera et al. (2017) and Seow et al. (2022) from the USA and Singapore, respectively, reported a reduction in falls, which is parallel to a finding from a systematic review of studies conducted in long-term settings (Mileski et al., 2019). Although the bed and chair alarm is a common strategy to reduce patient falls, nurses in the included studies expressed several challenges in terms of the distraction of their workflow and technical complexity (Balaguera et al., 2017; Fehlberg et al., 2020; Hoke & Zekany, 2020; King et al., 2018; Seow et al., 2022; Timmons et al., 2019). This conclusion aligns with world guidelines for fall prevention and several studies in the literature that highlight the limited effectiveness of bed and chair alarms as a single approach, which face challenges such as excessive noise that disturbs both nurses and patients, and the limited time staff have to respond to alerts, often leading to alarm fatigue (Ding et al., 2023; Montero-Odasso et al., 2022; Nyarko et al., 2024; Salameh et al., 2024). A cross-sectional observational study conducted by Staggs et al. (2020) reported that the use of bed and chair alarms did not lead to a reduction in patient falls— a finding that may be attributed to the inconsistent implementation of these alarms across various units. Therefore, nurses’ training regarding using this strategy is essential to improve the consistent application of bed and chair alarm (Mileski et al., 2019).

The current review underscores the importance of nurses’ assessment upon patient admission to identify their level of risk and tailor an effective fall prevention intervention based on individual needs. This aligns with findings from a systematic review, which concluded that assessment tools are consistently derived from established guidelines (Montero-Odasso et al., 2021).

Moreover, the studies included in this review employed various patient education approaches. The review revealed mixed effectiveness of these strategies in reducing fall incidence. Some studies reported a reduction in falls following patient education (Dykes et al., 2020; Guo et al., 2023; Klaiber et al., 2018). According to the World Fall Prevention Guidelines, this approach is advocated as a tailored method to reduce patient falls (Montero-Odasso et al., 2021). In addition, this result aligns with findings from a previous systematic review that enrolled geriatric patients from the community and any facility (Ojo & Thiamwong, 2022). In contrast, three studies that employed evidence-based approaches to fall prevention in acute care settings, such as the 6-PACK program, structured nurse-patient discussions, and motivational interviewing, found no significant reduction in fall rates (Barker et al., 2016; Kiyoshi-Teo et al., 2019; Weber et al., 2024). Research by Kiyoshi et al. (2019) in the USA and Weber et al. (2024) in Switzerland reported no fall reduction, which may be due to the small sample size, as the high-risk population restricted the applicability of the findings. Additionally, nurses encountered several obstacles when engaging in patient conversations, including background noise and the fast-paced clinical environment. Similarly, the study conducted in Australia by Barker et al. (2016) did not show a significant decrease in fall rates, likely due to the complexity of patients' conditions in acute care settings and the inconsistent application of strategies resulting from varying levels of nursing competency. This implies that the success of educational programs may vary depending on the complexity of acute care settings and patients’ condition.

Two studies included in this integrative review highlighted the importance of making certain environmental adjustments to improve the efficacy of fall prevention initiatives (Fehlberg et al., 2020; Innab, 2022). These findings were supported by a narrative review study that examined environmental changes aimed at preventing falls in patients, identifying several common strategies. These include maintaining the condition of floors and hallways and ensuring that call bells are readily accessible (Li & Surineni, 2025).

In the context of nurses’ training and education, nurses have acknowledged the positive effects of their training on falls prevention (Ayhan Oncu & Seren Intepeler, 2022; Innab, 2022; Ploeg et al., 2018; Radwan et al., 2024). A systematic review and a quasi-experimental study found that nurse training programs improve fall-related patient outcomes and enhance adherence to prevention strategies, highlighting the importance of ongoing professional development (Ojo & Thiamwong, 2022; Saki et al., 2023). However, this integrative review highlighted several challenges nurses face—such as scheduling conflicts and time constraints—that hinder their participation in training programs. Therefore, it is crucial to consider these barriers when developing fall prevention initiatives. Involving nurses in this process is essential, as their expertise and practical insights can significantly enhance program effectiveness.

In the context of enhancing the effectiveness of fall prevention programs, the role of administrative support is pivotal. Several studies in this review highlighted nurses’ emphasis on the importance of administrative support in ensuring the sustainability of fall prevention initiatives (Guo et al., 2023; Ploeg et al., 2018). Also, these studies have fostered multidisciplinary collaboration, which is crucial to address the complexities involved in fall prevention. This collaboration among various healthcare professionals can lead to more comprehensive and effective strategies, thereby improving patient outcomes and safety (Montero-Odasso et al., 2022). This result is consistent with Alvarado et al., (2024) who suggest that nurses should be empowered to take charge of practices that reduce and manage individual fall risks whenever feasible, with increased involvement from multidisciplinary teams as well as patients and their families.

### 3.1 Implications and recommendations for research and practice

This review highlights the programs and interventions based on evidence that nurses have implemented to prevent falls among adult patients during their hospital stays in acute care environments. These strategies are designed to improve patient safety and lower the likelihood of negative outcomes related to falls. As a result, this will contribute to a reduction in the frequency of falls and injuries associated with them, while also safeguarding the financial stability of the healthcare system, all while taking into account nurses' views on the effective fall prevention strategies based on their frontline experiences. Future research could consider performing a meta-analysis to assess the effectiveness of the fall prevention strategies that have been put into practice. Furthermore, future research may examine the cost-effectiveness of these strategies.

### 3.2 Strengths and limitations

This integrative review has several strong points, which are: (a) most of the studies were marked as high quality of 80% to 100%, (b) five different designs were included, which underscore the comprehensive synthesis of diverse evidence, (c) Eleven studies followed an evidence-based fall prevention program, and (d) The findings from this research conducted in various countries provide valuable insights for healthcare organizations worldwide to prevent patient falls a major global challenge in the healthcare sector, by enhancing fall prevention programs/strategies/interventions.

However, this study faced several limitations. First, the exclusion of non–full-text studies and dissertations, as well as the restriction to English-language publications, introduces potential selection and language bias. Second, some of the included studies had limited generalizability due to methodological factors such as sampling techniques, study design, and small sample sizes. Additionally, the variability in intervention types and outcome measures across studies made direct comparisons challenging. Furthermore, there is a potential for publication bias, as studies with non-significant results may be underrepresented in the literature. These limitations should be carefully considered when interpreting the findings and applying them to practice or policy. Future researchers should take the limitations as mentioned above into account when conducting an umbrella review or meta-analysis aimed at identifying the most effective interventions for reducing falls in acute care settings.

## 4 Conclusion

Falls in acute care settings pose a critical threat to patient safety, rehabilitation, and the overall effectiveness of healthcare outcomes. This study underscores the pivotal role that nurses play in fall prevention through diligent risk assessments, the development of individualized care plans, patient education, and collaboration with multidisciplinary teams. As primary healthcare providers, nurses are uniquely positioned to identify early signs of fall risk, implement targeted interventions, and foster a culture of safety within acute care environments. Their proactive involvement is essential in mitigating fall incidents and enhancing the quality of care delivered to patients. The analysis of this integrative review showed that integrating evidence-based strategies, training for nurses, and tailoring these interventions yielded promising results in decreasing patient falls. On the other hand, the analysis showed that using bed-chair alarms, individualized interventions, and patient education alone were not effective as a fall prevention strategy. This review underscores the importance of considering multiple fall prevention interventions led by nurses in acute care settings.

## Ethical statement

This study was conducted in accordance with ethical principles outlined in the Declaration of Helsinki. This review has been registered on the International Prospective Register of Systematic Reviews (PROSPERO CRD420251015159). Available from https://www.crd.york.ac.uk/PROSPERO/view/CRD420251015159.

## Data availability

The data supporting the findings of this study are available from the corresponding author [SA] upon reasonable request.

## Funding sources

This research did not receive any specific grant from funding agencies in the public, commercial, or not-for-profit sectors.

## CRediT authorship contribution statement

**Sahar Abdulkarim AlGhareeb:** Writing – review & editing, Writing – original draft, Visualization, Software, Methodology, Formal analysis, Data curation, Conceptualization. **Nora Ghalib AlOtaibi:** Writing – original draft, Software, Resources, Methodology, Formal analysis, Data curation. **Lujain Adel Sallam:** Writing – original draft, Resources, Methodology, Formal analysis, Data curation. **Adnan Innab:** Writing – review & editing, Visualization, Validation, Conceptualization.

## Declaration of competing interest

The authors declare that they have no known competing financial interests or personal relationships that could have appeared to influence the work reported in this article.

## Appendix Supplementary materials

Image, application 1
