# Supplementary material for: Nurse-led fall prevention programs in acute care settings: An integrative review
Source: Int J Nurs Stud Adv. 2025 Oct 25;9:100440. doi: 10.1016/j.ijnsa.2025.100440 (PMC12615328; doi:10.1016/j.ijnsa.2025.100440)
Supplement: Supplementary file 1 [file mmc1.docx]

**Table 1:** *Critical Appraisal of Qualitative Studies*

| **Author** | **Methodological quality criteria** | **Responses** | | | | **Total** |
| --- | --- | --- | --- | --- | --- | --- |
|  |  | **Yes** | **No** | **Can’t tell** | **Comment** | 100% |
| (Fehlberg et al., 2020) | S1. Are there clear research questions? | Yes |  |  |  |  |
|  | S2. Do the collected data allow to address the research questions? | Yes |  |  |  |  |
|  | 1.1. Is the qualitative approach appropriate to answer the research question? | Yes |  |  | because the study seeks to understand the nuanced, context-dependent decision-making processes of RNs. The Critical Decision Method (CDM) is particularly suitable for examining real-life, dynamic decision-making in clinical settings. |  |
|  | 1.2. Are the qualitative data collection methods adequate to address the research question? | Yes |  |  | Semistructured interviews based on the CDM were conducted, allowing for in-depth exploration of RNs' experiences and decision-making. The use of a 4-phase iterative process ensured comprehensive data collection. |  |
|  | 1.3. Are the findings adequately derived from the data? | Yes |  |  | The study identifies nine themes (e.g., fear of discipline, nurse judgment, unit culture) |  |
|  | 1.4. Is the interpretation of results sufficiently substantiated by data? | Yes |  |  | The authors provide detailed quotes and examples to illustrate each theme. Additionally, the discussion connects the findings to existing literature (e.g., bed alarm effectiveness, nurse morale) and offers practical recommendations. |  |
|  | 1.5. Is there coherence between qualitative data sources, collection, analysis and interpretation? | Yes |  |  | The study uses a consistent framework (RPDM-QHOM) to guide data collection and analysis. Peer debriefing and consensus processes further enhance the rigor of the analysis. |  |
| **Author** | **Methodological quality criteria** | **Responses** | | | | **Total** |
|  |  | **Yes** | **No** | **Can’t tell** | **Comment** | 100% |
| **(**Chad et al., 2024) | S1. Are there clear research questions? | Yes |  |  | Understand unit design features that may prevent falls and mitigate patient injuries. |  |
|  | S2. Do the collected data allow to address the research questions? | Yes |  |  | the qualitative data (walkthrough interviews, field notes, and photographs) directly address the research questions by capturing nurses' perceptions of environmental risks and design solutions. The study focuses on **unit layout, patient rooms, bathrooms, and nurses’ stations,** aligning with the goal of identifying fall risks in the built environment. |  |
|  | 1.1. Is the qualitative approach appropriate to answer the research question? | Yes |  |  | because the study seeks **subjective insights** from nurses about environmental hazards. |  |
|  | 1.2. Are the qualitative data collection methods adequate to address the research question? | Yes |  |  | **Walkthrough interviews** (conducted while touring units) enabled nurses to **point out specific hazards**(e.g., poor shower drainage, cluttered hallways).  **Semi-structured interviews**allowed flexibility in discussing unit design.  **Photographs and field notes** supplemented verbal data for richer analysis. | Triangulation |
|  | 1.3. Are the findings adequately derived from the data? | Yes |  |  | Direct quote, emeregent themes, trangulation |  |
|  | 1.4. Is the interpretation of results sufficiently substantiated by data? | Yes |  |  |  |  |
|  | 1.5. Is there coherence between qualitative data sources, collection, analysis and interpretation? | Yes |  |  | **Data sources**: Interviews, photos, and field notes.  **Collection**: Walkthroughs ensured real-time, context-rich feedback.  **Analysis**: Rapid assessment techniques and peer debriefing enhanced rigor.  **Interpretation**: Findings align with **participatory design principles** and practical safety improvements. |  |
| **Author** | **Methodological quality criteria** | **Responses** | | | | **Total** |
|  |  | **Yes** | **No** | **Can’t tell** | **Comment** | 100% |
| (Oncu & Intepeler, 2021) | S1. Are there clear research questions? | Yes |  |  | **Evaluate nurses’ views** on the implementation of evidence-based fall prevention interventions. |  |
|  | S2. Do the collected data allow to address the research questions? | Yes |  |  | Semi-structured with 17 nurses. The interviews explored nurses’ perceptions of training effectiveness, barriers, and suggestions for improvement, aligning with the study’s goals. |  |
|  | 1.1. Is the qualitative approach appropriate to answer the research question? | Yes |  |  | because the study seeks to understand **subjective experiences and perceptions** of nurses regarding fall prevention interventions |  |
|  | 1.2. Are the qualitative data collection methods adequate to address the research question? | Yes |  |  | **Semi-structured interviews** allowed for in-depth exploration of nurses’ views.  **Maximum variation sampling** ensured diverse perspectives (e.g., different departments, experience levels).  **Data saturation** was achieved (no new themes emerged after 15 interviews), ensuring comprehensive insights. |  |
|  | 1.3. Are the findings adequately derived from the data? | Yes |  |  | **Three main themes** (effectiveness of training, barriers, suggestions) emerged from thematic analysis.  Direct quotes and trangulation |  |
|  | 1.4. Is the interpretation of results sufficiently substantiated by data? | Yes |  |  | **Training effectiveness:** Nurses highlighted visual aids (posters, brochures) and social media as impactful (N4, N6). |  |
|  | 1.5. Is there coherence between qualitative data sources, collection, analysis and interpretation? | Yes |  |  | -Interviews with nurses from diverse departments. Standardized semi-structured questions ensured consistency  -Thematic analysis by two researchers, with peer debriefing to resolve discrepancies.  **-** Findings align with prior literature |  |
| **Author** | **Methodological quality criteria** | **Responses** | | | | **Total** |
|  |  | **Yes** | **No** | **Can’t tell** | **Comment** | 100 % |
| (Hoke & Zekany, 2020) | S1. Are there clear research questions? | Yes |  |  | To **describe and categorize**patient and nurse perspectives on falls.  To identify**nurses’ suggestions** for preventing falls. |  |
|  | S2. Do the collected data allow to address the research questions? | Yes |  |  | **Patient perspectives**: Why they believed they fell (e.g., slipping, dizziness).  **Nurse perspectives**: Why they believed patients fell (e.g., not calling for assistance).  **Prevention strategies**: Nurses’ suggestions (e.g., bed alarms, better education). |  |
|  | 1.1. Is the qualitative approach appropriate to answer the research question? | Yes |  |  | The study aims to uncover reasons behind falls, which quantitative data alone cannot capture. |  |
|  | 1.2. Are the qualitative data collection methods adequate to address the research question? | Yes |  |  | -Nurses interviewed patients using standardized questions  **-**Nurses documented patient/nurse perspectives and prevention suggestions.  **Triangulation**: Three independent coders analyzed themes to reduce bias. |  |
|  | 1.3. Are the findings adequately derived from the data? | Yes |  |  | Finding grounded in the data (3 main themes) |  |
|  | 1.4. Is the interpretation of results sufficiently substantiated by data? | Yes |  |  | -Patients-nurse disagreement  -Prevention strategies by nurses. |  |
|  | 1.5. Is there coherence between qualitative data sources, collection, analysis and interpretation? | Yes |  |  | -Standardized post-fall interviews and reflective emails.  -Content analysis by three coders, with themes validated through consensus. |  |
| **Author** | **Methodological quality criteria** | **Responses** | | | | **Total** |
|  |  | **Yes** | **No** | **Can’t tell** | **Comment** | 100% |
| (Baris & Intepeler, 2018). | S1. Are there clear research questions? | Yes |  |  | The study aims to explore the views and suggestions of healthcare professionals, patients, and family members on the causes of inpatient falls and fall-prevention practices. |  |
|  | S2. Do the collected data allow to address the research questions? | Yes |  |  | (semi-structured interviews)/ uses of thematic analysis  analyzed thematically and linked to the International Classification of Functioning, Disability and Health (ICF) framework |  |
|  | 1.1. Is the qualitative approach appropriate to answer the research question? | Yes |  |  | Qualitative methods are well-suited for capturing such nuanced data. |  |
|  | 1.2. Are the qualitative data collection methods adequate to address the research question? | Yes |  |  |  |  |
|  | 1.3. Are the findings adequately derived from the data? | Yes |  |  | The study uses thematic analysis to identify patterns and themes from the interview transcripts. The process is described in detail, including independent analysis by two researchers and consensus-building to ensure reliability. |  |
|  | 1.4. Is the interpretation of results sufficiently substantiated by data? | Yes |  |  | The linking of themes to the ICF framework further strengthens the interpretation. |  |
|  | 1.5. Is there coherence between qualitative data sources, collection, analysis and interpretation? | Yes |  |  | The design is consistent from data collection (semi-structured interviews with key stakeholders) to analysis (thematic analysis) and interpretation (linking themes to the ICF framework). |  |
| **Author** | **Methodological quality criteria** | **Responses** | | | | **Total** |
|  |  | **Yes** | **No** | **Can’t tell** | **Comment** | 100% |
| (King et al., 2016) | S1. Are there clear research questions? | Yes |  |  | The study aims to explore nurses' experiences with fall prevention in hospital settings and the impact of those experiences on how nurses provide care to fall-risk patients. |  |
|  | S2. Do the collected data allow to address the research questions? | Yes |  |  | (in-depth interviews and a focus group) and analysed using Grounded Dimensional Analysis (GDA), |  |
|  | 1.1. Is the qualitative approach appropriate to answer the research question? | Yes |  |  | The research seeks to understand nurses' experiences and perceptions, which requires an in-depth exploration of their perspective |  |
|  | 1.2. Are the qualitative data collection methods adequate to address the research question? | Yes |  |  | The iterative process of data collection and analysis in GDA further strengthens the methodology. |  |
|  | 1.3. Are the findings adequately derived from the data? | Yes |  |  | The study uses open, axial, and selective coding to analyze the data, and the process is described in detail. The conceptual model is developed based on the data, and participant quotes are provided to support the findings. |  |
|  | 1.4. Is the interpretation of results sufficiently substantiated by data? | Yes |  |  | The results are presented with direct quotes, themes, and conceptual model show the relationship |  |
|  | 1.5. Is there coherence between qualitative data sources, collection, analysis and interpretation? | Yes |  |  | The study design is consistent from data collection (interviews and focus group) to analysis (GDA) and interpretation (development of a conceptual model) |  |
| **Author** | **Methodological quality criteria** | **Responses** | | | | **Total** |
|  |  | **Yes** | **No** | **Can’t tell** | **Comment** | 100 % |
| (Timmons et al., 2019) | S1. Are there clear research questions? | Yes |  |  | The study aims to analyze the "failure" of a patient safety intervention (bed and bedside chair pressure sensors linked to radio pagers) using an agential realist framework. Specifically, it examines how the intervention and its outcomes are entangled with the sociomaterial context of the ward, including nurses, patients, and the RCT design. This is explicitly stated in the abstract and introduction.  It is a part of RCT, and contains crucial pieces of information |  |
|  | S2. Do the collected data allow to address the research questions? | Yes |  |  | The study combines qualitative data (interviews with nurses, HCAs, and patients, as well as structured observations) with insights from the RCT. |  |
|  | 1.1. Is the qualitative approach appropriate to answer the research question? | Yes |  |  | the qualitative approach is appropriate. The study seeks to understand the complex, context-dependent "failure" of a technological intervention, which requires an in-depth exploration |  |
|  | 1.2. Are the qualitative data collection methods adequate to address the research question? | Yes |  |  | Semi-structured interviews and structured observations provide rich, detailed insights into how the sensor system was used (or not used) in practice, |  |
|  | 1.3. Are the findings adequately derived from the data? | Yes |  |  | the findings are derived from the data. The study uses agential realism to analyze the qualitative data, focusing on the "intra-actions" between technology, humans, and the ward environment. |  |
|  | 1.4. Is the interpretation of results sufficiently substantiated by data? | Yes |  |  | The agential realist framework is applied consistently to explain how the RCT and sensor system "failed" due to their inability to account for the sociomaterial complexities of the ward. |  |
|  | 1.5. Is there coherence between qualitative data sources, collection, analysis and interpretation? | Yes |  |  | The study design aligns from data collection (interviews, observations) to analysis (agential realism) and interpretation (critique of RCT assumptions and technology design). The theoretical framework is used consistently to explain the findings, and the methodology is transparently reported. |  |

**Table 2:** *Critical Appraisal of Randomized Controlled Trials*

| **Author** | **Methodological quality criteria** | **Responses** | | | | **Total** |
| --- | --- | --- | --- | --- | --- | --- |
|  |  | **Yes** | **No** | **Can’t tell** | **Comment** | 80% |
| (Klaiber et al., 2018) | S1. Are there clear research questions? | Yes |  |  |  |  |
|  | S2. Do the collected data allow to address the research questions? | Yes |  |  |  |  |
|  | 2.1. Is randomization appropriately performed? | Yes |  |  | The study used cluster randomization, where hospital wards were randomly assigned to either the intervention or control group. |  |
|  | 2.2. Are the groups comparable at baseline? | Yes |  |  | The baseline characteristics of the patients, including sex, age, body mass index, American Society of Anaesthesiologists (ASA) score, target organ for operation, comorbidities, and duration of operation. |  |
|  | 2.3. Are there complete outcome data? | Yes |  |  | The missing data was excluded from the data analysis. |  |
|  | 2.4. Are outcome assessors blinded to the intervention provided? |  | No |  | The article does not explicitly state whether the outcome assessors were blinded to the intervention provided. |  |
|  | 2.5 Did the participants adhere to the assigned intervention? | Yes |  |  | Most participants adhered to the assigned intervention, but there were some deviations. In the intervention group, 24.6% of patients did not attend the preoperative seminar, mainly due to competing appointments for preoperative preparations. |  |
| **Author** | **Methodological quality criteria** | **Responses** | | | | **Total** |
|  |  | **Yes** | **No** | **Can’t tell** | **Comment** | 100% |
| (Barker et al., 2016) | S1. Are there clear research questions? | Yes |  |  | to evaluate the effect of the 6-PACK programme on falls and fall injuries in acute hospital wards. |  |
|  | S2. Do the collected data allow to address the research questions? | Yes |  |  |  |  |
|  | 2.1. Is randomization appropriately performed? | Yes |  |  | The study used a cluster randomized controlled design. hen randomly assigned to intervention (6-PACK) or control (usual care) groups. A statistician blinded to ward identities generated the allocation sequence using Stata, ensuring concealment |  |
|  | 2.2. Are the groups comparable at baseline? | Yes |  |  | The groups were comparable at baseline. shows similar patient demographics. |  |
|  | 2.3. Are there complete outcome data? | Yes |  |  | No loss to follow-up was reported. |  |
|  | 2.4. Are outcome assessors blinded to the intervention provided? | Yes |  |  | Partially, nurses and patients could not be blinded due to the nature of the intervention, independent assessors coding fall characteristics and injuries were blinded to group allocation. The primary statistician analyzing the data was also blinded. However, assessors collecting process data (e.g., adherence to 6-PACK components) were unblinded, which could introduce detection bias for process measures but not for the primary outcomes. |  |
|  | 2.5 Did the participants adhere to the assigned intervention? | Yes |  |  | adherence was high |  |
| **Author** | **Methodological quality criteria** | **Responses** | | | | **Total** |
|  |  | **Yes** | **No** | **Can’t tell** | **Comment** | 60% |
| (Kiyoshi-Teo et al., 2019) | S1. Are there clear research questions? | Yes |  |  | -Evaluate the **feasibility** of a brief motivational interviewing (MI) intervention for fall prevention in hospitalized older adults (e.g., recruitment/retention rates, intervention fidelity).  -Explore the **potential efficacy** of MI in changing fall-related perceptions (e.g., fear of falling) and behaviors (e.g., adherence to prevention strategies). |  |
|  | S2. Do the collected data allow to address the research questions? | Yes |  |  |  |  |
|  | 2.1. Is randomization appropriately performed? | Yes |  |  | Participants were randomized in blocks of 10 to ensure balance across three hospital units. A computer-generated sequence was used, and while blinding was not feasible for participants or providers, the method minimized selection bias. |  |
|  | 2.2. Are the groups comparable at baseline? | Yes |  |  | No significant differences were found in demographics, fall risk (Morse Fall Scale), cognitive status (MoCA-B), fall history, or baseline measures of fear of falling (FESI-S), behaviors (M-FaB), or patient activation (PAM). |  |
|  | 2.3. Are there complete outcome data? |  | No |  | Only 66% (67) patients completed the trail, Reasons included death, withdrawal, and inability to contact. However, the attrition rate (34%) may bias long-term results if dropouts differed systematically. |  |
|  | 2.4. Are outcome assessors blinded to the intervention provided? |  | No |  | outcome assessors were not blinded. The principal investigator delivered the MI intervention and collected data, introducing potential bias. |  |
|  | 2.5 Did the participants adhere to the assigned intervention? | Yes |  |  | adherence was high |  |

**Table 3:** *Critical Appraisal of Non-Randomized Control Trials*

| **Author** | **Methodological quality criteria** | **Responses** | | | | **Total** |
| --- | --- | --- | --- | --- | --- | --- |
|  |  | **Yes** | **No** | **Can’t tell** | **Comment** | 100% |
| (Dykes et al., 2020) | S1. Are there clear research questions? | Yes |  |  |  |  |
|  | S2. Do the collected data allow to address the research questions? | Yes |  |  |  |  |
|  | 3.1. Are the participants representative of the target population? | Yes |  |  | Large sample size (3 medical centers)  3 medical center, 14 wards |  |
|  | 3.2. Are measurements appropriate regarding both the outcome and intervention (or exposure)? | Yes |  |  |  |  |
|  | 3.3. Are there complete outcome data? | Yes |  |  |  |  |
|  | 3.4. Are the confounders accounted for in the design and analysis? | Yes |  |  | As mentioned by the authors, problem analysis,design,development, pilot implementation, and evaluation periods were inserted into the interrupted time series analysis to account for potential confounders associated with developing the intervention. (Figure 2. P3) |  |
|  | 3.5. During the study period, is the intervention administered (or exposure occurred) as intended? | Yes |  |  |  |  |
| **Author** | **Methodological quality criteria** | **Responses** | | | | **Total** |
|  |  | **Yes** | **No** | **Can’t tell** | **Comment** | 60% |
| (Guo et al., 2022) | S1. Are there clear research questions? | Yes |  |  | The questions were not explicily addressed, however there was a clear aim. |  |
|  | S2. Do the collected data allow to address the research questions? | Yes |  |  |  |  |
|  | 3.1. Are the participants representative of the target population? |  | No |  | However, limited to single center might limit the generalizability (58 SZ)  Additionally, small sample size, limited time, lack of randomization, all of these factors limit the generalizability as mentioned in the limitation. |  |
|  | 3.2. Are measurements appropriate regarding both the outcome and intervention (or exposure)? | Yes |  |  |  |  |
|  | 3.3. Are there complete outcome data? | Yes |  |  |  |  |
|  | 3.4. Are the confounders accounted for in the design and analysis? |  | No |  |  |  |
|  | 3.5. During the study period, is the intervention administered (or exposure occurred) as intended? | Yes |  |  |  |  |
| **Author** | **Methodological quality criteria** | **Responses** | | | | **Total** |
|  |  | **Yes** | **No** | **Can’t tell** | **Comment** | 100% |
| (Seow et al., 2021) | S1. Are there clear research questions? | Yes |  |  | The questions were not explicitly addressed, however there was a clear aim. |  |
|  | S2. Do the collected data allow to address the research questions? | Yes |  |  |  |  |
|  | 3.1. Are the participants representative of the target population? | Yes |  |  | As mentioned in the limitation, although it was a single center, but it is serving a high segment of the population, and the selected wards had a very high discharge rate. Additionally, the authors collected all the eligible participants during the study period (census method). Moreover, the consideration of including confounders in the analysis. All these factors to overcome the single center issue and to enhance the external validity. |  |
|  | 3.2. Are measurements appropriate regarding both the outcome and intervention (or exposure)? | Yes |  |  |  |  |
|  | 3.3. Are there complete outcome data? | Yes |  |  | Those were excluded because they did not meet the inclusion criteria, they should not be in the study. They are were not included in the analysis (study design P.2). the author did not report any missing data which indicate complete data from the all included participants during the study period. |  |
|  | 3.4. Are the confounders accounted for in the design and analysis? | Yes |  |  | Covariates were evaluated. |  |
|  | 3.5. During the study period, is the intervention administered (or exposure occurred) as intended? | Yes |  |  | The authors explicilty mentioned that the decision of the alarm will depend on the nurses’ decision about the case. The intervention was conducted as intended.  (intervention P.2). |  |
| **Author** | **Methodological quality criteria** | **Responses** | | | | **Total** |
|  |  | **Yes** | **No** | **Can’t tell** | **Comment** | 60% |
| (Balaguera et al., 2017) | S1. Are there clear research questions? | Yes |  |  | The study did not explicitly mention a research question. The aim was to conduct a technology evaluation, including feasibility, usability, and user experience, of a medical  sensor-based Intranet of things (IoT) system in facilitating nursing response to bed exits in an acute care hospital |  |
|  | S2. Do the collected data allow to address the research questions? | Yes |  |  |  |  |
|  | 3.1. Are the participants representative of the target population? |  | No |  | The sample is from a single hospital, which may limit generalizability. |  |
|  | 3.2. Are measurements appropriate regarding both the outcome and intervention (or exposure)? | Yes |  |  | The study uses objective fall monitoring data, making the measurements reliable, and they assessed user experience via nurse focus groups.  Qualitative data, but there is no control group. |  |
|  | 3.3. Are there complete outcome data? | Yes |  |  |  |  |
|  | 3.4. Are the confounders accounted for in the design and analysis? |  | No |  | The study does not appear to control for external factors that might influence fall rates, such as staffing levels or patient conditions. |  |
|  | 3.5. During the study period, is the intervention administered (or exposure occurred) as intended? | Yes |  |  | The article describes the implementation of the system as planned, without deviations. |  |
| **Author** | **Methodological quality criteria** | **Responses** | | | | **Total** |
|  |  | **Yes** | **No** | **Can’t tell** | **Comment** | 80% |
| (Cerilo & Siegmund, 2021) | S1. Are there clear research questions? | Yes |  |  | Examine the effect of a **nurse-led multimodal intervention** (video education + reinforcement) on **fall risk awareness, self-efficacy, and engagement**in hospitalized older adults. |  |
|  | S2. Do the collected data allow to address the research questions? | Yes |  |  | **Data adequately addressed** the primary question (fall risk awareness) and some secondary hypotheses (self-efficacy-engagement, mobility/polypharmacy effects). |  |
|  | 3.1. Are the participants representative of the target population? |  | No |  | Convenience sampling limit the generalizability |  |
|  | 3.2. Are measurements appropriate regarding both the outcome and intervention (or exposure)? | Yes |  |  | Validated tools were used |  |
|  | 3.3. Are there complete outcome data? | Yes |  |  | 60 participants were completed the trail |  |
|  | 3.4. Are the confounders accounted for in the design and analysis? | Yes |  |  | **Controlled for health literacy** (all participants scored adequately).  **Used ANCOVA** to adjust for baseline scores.  **Tested sub-variables**(e.g., depression, mobility aids) via t-tests/correlations. |  |
|  | 3.5. During the study period, is the intervention administered (or exposure occurred) as intended? | Yes |  |  | **Video education**: 10-minute standardized video.  **Nurse reinforcement**: Individualized teach-back sessions.  **Fidelity:** Nurse researcher ensured consistency; teach-back addressed knowledge gaps. |  |

**Table 4:** *Critical Appraisal of Quantitative Descriptive Study*

| **Author** | **Methodological quality criteria** | **Responses** | | | | **Total** |
| --- | --- | --- | --- | --- | --- | --- |
|  |  | **Yes** | **No** | **Can’t tell** | **Comment** | 60% |
| (Innab, 2022) | S1. Are there clear research questions? | Yes |  |  |  |  |
|  | S2. Do the collected data allow to address the research questions? | Yes |  |  |  |  |
|  | 4.1. Is the sampling strategy relevant to address the research question? | Yes |  |  |  |  |
|  | 4.2. Is the sample representative of the target population? |  | No |  | The researcher used a convenience  sampling method & single center, reducing the generalizability of the findings to  other healthcare settings. |  |
|  | 4.3. Are the measurements appropriate? | Yes |  |  |  |  |
|  | 4.4. Is the risk of nonresponse bias low? |  | No |  | The study does not mention how non-respondents might differ from respondents, making nonresponse bias a concern. The author mentions the (response  rate = 68%). |  |
|  | 4.5. Is the statistical analysis appropriate to answer the research question? | Yes |  |  | Descriptive statistics and t-test were used appropriately to analyze survey responses. |  |
| **Author** | **Methodological quality criteria** | **Responses** | | | | **Total** |
|  |  | **Yes** | **No** | **Can’t tell** | **Comment** | 60% |
| (Dykes et al., 2021) | S1. Are there clear research questions? | Yes |  |  | The study did not explicitly mention a research question. But The study explicitly aims to evaluate the effectiveness of the FallTIPS program using a perceived efficacy tool. |  |
|  | S2. Do the collected data allow to address the research questions? | Yes |  |  | Random sampling. |  |
|  | 4.1. Is the sampling strategy relevant to address the research question? | Yes |  |  |  |  |
|  | 4.2. Is the sample representative of the target population? |  |  | Can’t tell | According to authors To maximize the response rate to assure representativeness of a small population, they followed Dillman's recommendations of sending several requests for participation, and planned for 3-weeks of data collection. However, the authors acknowledged these limitations, noting that the results may not be representative of all hospitals and non-surgical units. Additional FPES research is needed with larger and more diverse samples. |  |
|  | 4.3. Are the measurements appropriate? | Yes |  |  | The study uses validated tools to measure perceived efficacy and fall prevention outcomes. |  |
|  | 4.4. Is the risk of nonresponse bias low? |  |  | Can’t tell | 52.7%  participation rate. |  |
|  | 4.5. Is the statistical analysis appropriate to answer the research question? | Yes |  |  | The study describes proper implementation of the FallTIPS program as planned. |  |
| **Author** | **Methodological quality criteria** | **Responses** | | | | **Total** |
|  |  | **Yes** | **No** | **Can’t tell** | **Comment** | 80% |
| (Wyss-Hänecke et al., 2023) | S1. Are there clear research questions? | Yes |  |  | The study did not explicitly mention a research question. But the study aims to identify ward-level system factors associated with the implementation fidelity of a multifactorial fall prevention program (StuPA) in an acute care hospital setting. Specifically, the study seeks to understand how factors such as patient care dependency and the number of patient transfers influence the fidelity of the fall prevention program. |  |
|  | S2. Do the collected data allow to address the research questions? | Yes |  |  | The study uses two main data sources: administrative data from patient records and a survey evaluating the implementation fidelity of the StuPA program. These data sources provide information on patient characteristics (e.g., care dependency, number of transfers) and the fidelity of the fall prevention program, which are directly relevant to the research questions. |  |
|  | 4.1. Is the sampling strategy relevant to address the research question? |  |  | Can’t tell | The study includes data from 19 acute care wards at the University Hospital Basel, Switzerland, focusing on adult patients admitted between July and December 2019. |  |
|  | 4.2. Is the sample representative of the target population? | Yes |  |  | The census method was used to recruit all eligible participants during the study period, the study was cross-sectional multi-centre. Although the authors did not provide information about if these patents characteristics represent population, using multi-canters enhance the external validity that provide generalizability. |  |
|  | 4.3. Are the measurements appropriate? | Yes |  |  | The measurements are appropriate. The study uses validated tools and methods, such as the ePA-AC scale for care dependency and a structured survey to assess implementation fidelity. The variables chosen (e.g., care dependency, number of transfers, fall rates) are relevant to the research questions and are measured using reliable and valid instruments |  |
|  | 4.4. Is the risk of nonresponse bias low? | Yes |  |  | The survey data were 100% complete. |  |
|  | 4.5. Is the statistical analysis appropriate to answer the research question? | Yes |  |  | The study uses descriptive statistics, Pearson’s correlation coefficients, and linear regression modelling to analyse the data. These methods are suitable for identifying associations between ward-level system factors. |  |
| **Author** | **Methodological quality criteria** | **Responses** | | | | **Total** |
|  |  | **Yes** | **No** | **Can’t tell** | **Comment** | 60% |
| (Staggs et al., 2020) | S1. Are there clear research questions? | Yes |  |  | Prevalence of bed/chair alarm use in US hospitals. Identify patient- and unit-level factors associated with alarm use.  Estimate the variability in alarm use attributable to patient- versus unit-level factors. |  |
|  | S2. Do the collected data allow to address the research questions? |  |  |  |  |  |
|  | 4.1. Is the sampling strategy relevant to address the research question? | Yes |  |  | the study used a stratified random sampling approach |  |
|  | 4.2. Is the sample representative of the target population? |  | No |  | Only NDNQI-member hospitals were included, which may differ from non-member hospitals in resources or practices.  Two-thirds of units were medical or medical-surgical, limiting generalizability to surgical units. |  |
|  | 4.3. Are the measurements appropriate? | Yes |  |  |  |  |
|  | 4.4. Is the risk of nonresponse bias low? | Yes |  |  | Minimal bias |  |
|  | 4.5. Is the statistical analysis appropriate to answer the research question? | Yes |  |  | **Logistic mixed models**  **Propensity scores**  **Model comparisons**  **Multiple imputation** |  |
| **Author** | **Methodological quality criteria** | **Responses** | | | | **Total** |
|  |  | **Yes** | **No** | **Can’t tell** | **Comment** | 80% |
| (Vechter et al., 2024) | S1. Are there clear research questions? | Yes |  |  | Hypothesis |  |
|  | S2. Do the collected data allow to address the research questions? | Yes |  |  |  |  |
|  | 4.1. Is the sampling strategy relevant to address the research question? | Yes |  |  |  |  |
|  | 4.2. Is the sample representative of the target population? |  | No |  | Convenience sampling was collected from one setting |  |
|  | 4.3. Are the measurements appropriate? | Yes |  |  |  |  |
|  | 4.4. Is the risk of nonresponse bias low? | Yes |  |  |  |  |
|  | 4.5. Is the statistical analysis appropriate to answer the research question? | Yes |  |  |  |  |

**Table 5:** *Critical Appraisal of Mixed-Methods Study*

| **Author** | **Methodological quality criteria** | **Responses** | | | | **Total** |
| --- | --- | --- | --- | --- | --- | --- |
|  |  | **Yes** | **No** | **Can’t tell** | **Comment** | 60% |
| (Weber, K.et al., 2024) | S1. Are there clear research questions? | Yes |  |  | assumption and objective were well stated. |  |
|  | S2. Do the collected data allow to address the research questions? | Yes |  |  |  |  |
|  | 5.1. Is there an adequate rationale for using a mixed methods design to address the research question? | Yes |  |  |  |  |
|  | 5.2. Are the different components of the study effectively integrated to answer the research question? |  |  | Can’t tell | Partially effective (quantitative positive pt. experience) (qualitative revealed barriers to implementation) |  |
|  | 5.3. Are the outputs of the integration of qualitative and quantitative components adequately interpreted? |  |  | Can’t tell | The interpretation of the results is partially adequate |  |
|  | 5.4. Are divergences and inconsistencies between quantitative and qualitative results adequately addressed? | Yes |  |  | The study acknowledges the apparent discrepancy |  |
|  | 5.5. Do the different components of the study adhere to the quality criteria of each tradition of the methods involved? | Yes |  |  |  |  |
| **Author** | **Methodological quality criteria** | **Responses** | | | | **Total** |
|  |  | **Yes** | **No** | **Can’t tell** | **Comment** | 80% |
| (Vechter & Drach‐Zahavy, 2021) | S1. Are there clear research questions? | Yes |  |  | Understand the distinctive experiences and strategies of high- and low-resilience nurses in preventing patient falls. |  |
|  | S2. Do the collected data allow to address the research questions? | Yes |  |  |  |  |
|  | 5.1. Is there an adequate rationale for using a mixed methods design to address the research question? | Yes |  |  | Quan/Qual This combination allows for a comprehensive understanding of how resilience translates into fall prevention strategies, aligning with the study's aim to explore both measurable traits and contextual behaviors. |  |
|  | 5.2. Are the different components of the study effectively integrated to answer the research question? | Yes |  |  | Quantitative grouping informed qualitative analysis (e.g., comparing themes between high- and low-resilience nurses). Thematic findings (scepticism, anticipation, proactivity) were directly linked to resilience levels. |  |
|  | 5.3. Are the outputs of the integration of qualitative and quantitative components adequately interpreted? | Yes |  |  | The study clearly interprets how quantitative resilience scores correlate with qualitative themes |  |
|  | 5.4. Are divergences and inconsistencies between quantitative and qualitative results adequately addressed? |  | No |  | The study does not report significant divergences between quantitative and qualitative results. (findings consistently aligned) |  |
|  | 5.5. Do the different components of the study adhere to the quality criteria of each tradition of the methods involved? | Yes |  |  | **-Reliability & Validity**: The BRS is a validated scale (α = 0.72 in this study), supporting its reliability.  **-**Purposive sampling was used (N=24), which is acceptable for exploratory studies but limits generalizability. |  |
| **Author** | **Methodological quality criteria** | **Responses** | | | | **Total** |
|  |  | **Yes** | **No** | **Can’t tell** | **Comment** | 60% |
| (Balaguera et al., 2017) | S1. Are there clear research questions? | Yes |  |  | **Primary objective**: Evaluate the feasibility, usability, and user experience of a medical IoT system (SensableCare) for preventing bed falls in acute care.  **Secondary focus**: Assess nurse response times to bed-exit alerts and system integration into clinical workflows. |  |
|  | S2. Do the collected data allow to address the research questions? | Yes |  |  |  |  |
|  | 5.1. Is there an adequate rationale for using a mixed methods design to address the research question? | Yes |  |  | Quantify system performance (response times, PPV).  Qualitatively explore nurse experiences (barriers, facilitators).  Triangulate findings (e.g., fast response times + positive nurse feedback). |  |
|  | 5.2. Are the different components of the study effectively integrated to answer the research question? |  |  | Cant tell | No explicit "joint display" linking quantitative and qualitative results |  |
|  | 5.3. Are the outputs of the integration of qualitative and quantitative components adequately interpreted? | Yes |  |  | Links fast response times (quantitative) to nurses’ positive feedback (qualitative).  Attributes slower overnight responses to staffing challenges (qualitative explanation for quantitative finding).  Acknowledges limitations (e.g., Hawthorne effect, concurrent use of legacy alarms. |  |
|  | 5.4. Are divergences and inconsistencies between quantitative and qualitative results adequately addressed? | No |  |  |  |  |
|  | 5.5. Do the different components of the study adhere to the quality criteria of each tradition of the methods involved? | Yes |  |  | Appropriate statistical analysis  Thematic analysis of nurse feedback.  Saturation likely achieved (25 nurses across 2 sessions). |  |
| **Author** | **Methodological quality criteria** | **Responses** | | | | **Total** |
|  |  | **Yes** | **No** | **Can’t tell** | **Comment** | 100% |
| (Ploeg et al., 2018) | S1. Are there clear research questions? | Yes |  |  |  |  |
|  | S2. Do the collected data allow to address the research questions? | Yes |  |  |  |  |
|  | 5.1. Is there an adequate rationale for using a mixed methods design to address the research question? | Yes |  |  | Both qualitative and quantitative data were collected to understand  the sustainability of the guideline implementation |  |
|  | 5.2. Are the different components of the study effectively integrated to answer the research question? | Yes |  |  |  |  |
|  | 5.3. Are the outputs of the integration of qualitative and quantitative components adequately interpreted? | Yes |  |  |  |  |
|  | 5.4. Are divergences and inconsistencies between quantitative and qualitative results adequately addressed? | Yes |  |  | With respect to staff and their confidence in their  involvement and training to sustain the process of implementation  of the fall prevention guideline, while the  scores started very high (92.46%), they did decline  (70.58%), although not statistically significantly.  Organizationally, participant scores on the investments  in infrastructure made by all three of the hospital sites  increased, although not statistically significant, from  68.02% in the intervention period to 79.53% in the postintervention  period. |  |
|  | 5.5. Do the different components of the study adhere to the quality criteria of each tradition of the methods involved? | Yes |  |  |  |  |
